# Intraneuronal tau aggregation induces the integrated stress response in astrocytes

**DOI:** 10.1093/jmcb/mjac071

**Published:** 2022-12-15

**Authors:** Kevin L Batenburg, Nael N Kasri, Vivi M Heine, Wiep Scheper

**Affiliations:** Department of Functional Genomics, Center for Neurogenomics and Cognitive Research, Amsterdam Neuroscience, Vrije Universiteit Amsterdam, De Boelelaan 1085, 1081 HV Amsterdam, The Netherlands; Department of Human Genetics and Cognitive Neuroscience, Donders Institute for Brain, Cognition and Behaviour, Radboudumc, Geert Grooteplein 10 Noord, 6500 HB Nijmegen, The Netherlands; Department of Child and Adolescent Psychiatry, Amsterdam UMC location Vrije Universiteit, Amsterdam Neuroscience, Vrije Universiteit Amsterdam, De Boelelaan 1085, 1081 HV Amsterdam, The Netherlands; Department of Complex Trait Genetics, Center for Neurogenomics and Cognitive Research, Amsterdam Neuroscience, Vrije Universiteit Amsterdam, De Boelelaan 1085, 1081 HV Amsterdam, The Netherlands; Department of Functional Genomics, Center for Neurogenomics and Cognitive Research, Amsterdam Neuroscience, Vrije Universiteit Amsterdam, De Boelelaan 1085, 1081 HV Amsterdam, The Netherlands; Department of Human Genetics, Amsterdam UMC location Vrije Universiteit, De Boelelaan 1085, 1081 HV Amsterdam, The Netherlands

**Keywords:** tau aggregation, astrocytes, hiPSC-derived neurons, integrated stress response, oxidative stress, antisense oligonucleotides

## Abstract

Progressive aggregation of tau protein in neurons is associated with neurodegeneration in tauopathies. Cell non-autonomous disease mechanisms in astrocytes may be important drivers of the disease process but remain largely elusive. Here, we studied cell type-specific responses to intraneuronal tau aggregation prior to neurodegeneration. To this end, we developed a fully human co-culture model of seed-independent intraneuronal tau pathology, which shows no neuron and synapse loss. Using high-content microscopy, we show that intraneuronal tau aggregation induces oxidative stress accompanied by activation of the integrated stress response specifically in astrocytes. This requires the direct co-culture with neurons and is not related to neurodegeneration or extracellular tau levels. Tau-directed antisense therapy reduced intraneuronal tau levels and aggregation and prevented the cell non-autonomous responses in astrocytes. These data identify the astrocytic integrated stress response as a novel disease mechanism activated by intraneuronal tau aggregation. In addition, our data provide the first evidence for the efficacy of tau-directed antisense therapy to target cell autonomous and cell non-autonomous disease pathways in a fully human model of tau pathology.

## Introduction

Aggregation of tau protein is a major pathological hallmark of neurodegenerative tauopathies, including Alzheimer's disease (AD) and frontotemporal dementias (FTD) (Spillantini and Goedert, 2013). Tau pathology strongly correlates with neurodegeneration and clinical symptoms, demonstrated in post- (Gómez-Isla et al., 1997) and antemortem studies (Ossenkoppele et al., 2016). Therefore, therapeutics that target tau expression, hyperphosphorylation, seeding, and/or aggregation present a much investigated and promising approach for therapeutic intervention in tauopathies (Congdon and Sigurdsson, 2018). Rodent *in vitro* and *in vivo* models of tau pathology have been valuable tools for target identification and therapeutic intervention, but have limited translational value to human tauopathy (Drummond and Wisniewski, 2017). As a result, the underlying disease mechanisms of neurodegeneration remain largely unknown. Moreover, the absence of pre-clinical evidence for the efficacy of tau-directed therapeutics in human context may be an important reason for failure in clinical trials.

Since tau is abundantly expressed in neurons where it predominantly accumulates in disease conditions, tau aggregation can disrupt neuronal physiology in a cell-autonomous manner (Götz et al., 2019). In addition, glial cells are affected by neuronal tau pathology in a cell non-autonomous manner (Leyns and Holtzman, 2017). Astrocytes are tightly integrated in the neural network where they perform key homeostatic functions (Verkhratsky and Nedergaard, 2018) and closely associate with neurons containing tau aggregates (Bouvier et al., 2016). Moreover, astrocytes are increasingly recognized as important contributors to neurodegeneration via a loss of homeostatic function and/or gain of toxic function (Hasel et al., 2017; Sidoryk-Wegrzynowicz et al., 2017; Reid et al., 2020). However, it is currently unknown whether this is caused by neuronal tau aggregation, and whether it can be modulated by tau-directed therapeutic intervention. Human induced pluripotent stem cell (hiPSC) technology has recently created the possibility to study such disease mechanisms in human neurons and astrocytes in a translationally relevant *in vitro* context. Indeed, hiPSC-derived neurons are increasingly employed to study tau pathology (Ehrlich et al., 2015; Iovino et al., 2015; Sposito et al., 2015; Verheyen et al., 2015; Esteras et al., 2017), but so far inefficiently develop insoluble tau aggregates spontaneously (Ehrlich et al., 2015; Iovino et al., 2015; Sposito et al., 2015) which has necessitated the use of extracellular seeds to induce intraneuronal aggregation (Verheyen et al., 2015). Seeds, however, can elicit responses in astrocytes independently of intraneuronal tau aggregates (Ungerleider et al., 2021; Wang and Ye, 2021) and are therefore a confounding factor in the study of cell (non-)autonomous disease mechanisms.

Here we established and extensively validated the first fully human neuron–astrocyte co-culture displaying seed-independent, progressive intraneuronal tau aggregation, to model the cell (non-) autonomous disease mechanisms of tau pathology more accurately. Using this model, we identified oxidative stress and activation of the integrated stress response (ISR) in astrocytes as an early, cell non-autonomous response to tau aggregation prior to neurodegeneration that can be blocked by a tau-targeting therapeutic.

## Results

### Development and validation of a human neuron–astrocyte co-culture model of seed-independent intraneuronal tau aggregation

**Figure 1:**
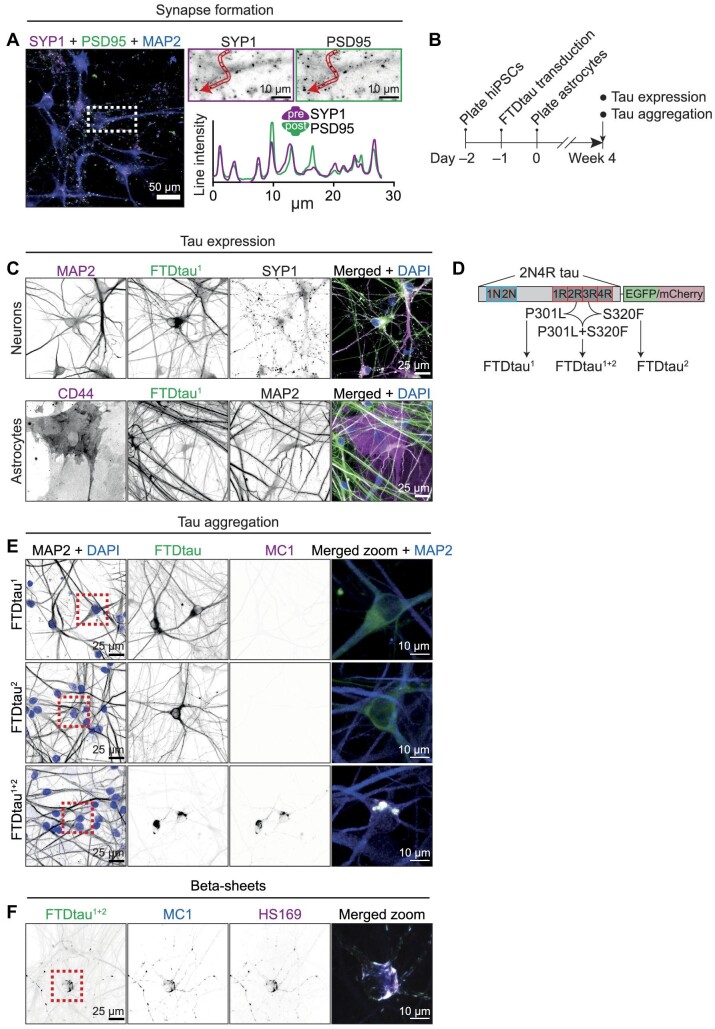
Intraneuronal tau aggregation in human neuron–astrocyte co-culture. (**A**) A representative confocal image of neuronal maturation in human neuron–astrocyte co-culture at 4 weeks. Immunostaining was performed for dendrites (MAP2, blue), presynapses (SYP1, magenta), and postsynapses (PSD95, green). In a zoom of the boxed area indicated in the merged image, the SYP1 and PSD95 single channels are shown in greyscale. Intensity profiles of SYP1 (magenta) and PSD95 (green) were determined along the line segment indicated in the single channels to show co-localization of pre- and postsynaptic compartments. (**B**) Schematic representation of the protocol to introduce intraneuronal tau pathology in human neuron–astrocyte co-culture. Ngn2-hiPSCs are plated at experimental Day –2 and virally transduced with the different FTDtau constructs on Day –1 before plating of astrocytes at Day 0. Tau aggregation was assessed after 4 weeks. See Materials and methods for more detail. (**C**) Representative confocal images show FTDtau^1^ expression in neurons but not astrocytes. Co-cultures were transduced with EGFP-tagged FTDtau^1^ and subsequently immunostained for MAP2 and SYP1 or CD44 at Week 4. Cell nuclei were visualized by DAPI. Single channels are shown in greyscale. Merged images include MAP2 or CD44 (magenta), FTDtau^1^ (green), SYP1 or MAP2 (greyscale), and DAPI (blue). (**D**) EGFP- or mCherry-tagged 2N4R tau mutants used in this study, containing the P301L (FTDtau^1^), S320F (FTDtau^2^), or P301L + S320F (FTDtau^1+2^) mutations. (**E**) Representative confocal images of tau aggregation in neuron–astrocyte co-culture at 4 weeks, transduced with EGFP-tagged FTDtau^1^, FTDtau^2^, or FTDtau^1+2^. EGFP direct fluorescence and immunostainings for MAP2 and a pathological conformation of tau (MC1) are shown in greyscale. Cell nuclei were visualized using DAPI (blue). Zooms of the boxed area indicated in the MAP2 + DAPI panels are shown as merged image of MAP2 (blue), FTDtau (green), and MC1 (magenta). (**F**) Representative confocal images of a neuron–astrocyte co-culture expressing EGFP-FTDtau^1+2^, immunostained for pathological tau (MC1), and co-labelled for stacked beta-sheets (HS169). Single channels are shown in greyscale. A zoom of the boxed area indicated in the FTDtau^1+2^ channel is shown as merged image and shows overlap of FTDtau^1+2^ (green), MC1 (blue), and HS169 (magenta).

Efficient generation of predominantly excitatory neurons from hiPSCs was previously achieved by expression of neurogenin 2 (Ngn2) (Zhang et al., 2013). We therefore employed a clonal hiPSC-line with doxycyclin-inducible Ngn2 (Frega et al., 2017) and adjusted the protocol for co-culture with primary human astrocytes. Confocal imaging demonstrated that after 4 weeks MAP2-positive neurons with co-localizing presynaptic synaptophysin 1 (SYP1) and postsynaptic density protein 95 (PSD95) puncta were generated (Figure 1A), confirming that neurons in these co-cultures form a morphologically mature synapse pattern.

Tau is mainly expressed in neurons where it predominantly accumulates in disease conditions. To selectively introduce tau in neurons in the co-culture model, viral transduction with human 2N4R tau containing the P301L mutation (FTDtau^1^) was performed 1 day before the addition of human astrocytes (Figure 1B). Confocal microscopy showed FTDtau^1^ expression in MAP2- and SYP1-positive neurons, but not in CD44-positive astrocytes (Figure 1C), demonstrating neuron-specific FTDtau^1^ expression. Analysis of FTDtau^1^-transduced and untransduced co-cultures by high-content microscopy and automated analysis (Supplementary Figures S1 and S2A) showed that neuronal viability, morphology, and presynapses were unaffected by FTDtau^1^-overexpression (Supplementary Figure S2B–I). Therefore, we established a transduction strategy that results in neuron-specific FTDtau expression without affecting neuronal morphology and viability.

In agreement with previous reports FTDtau^1^ expression does not spontaneously form aggregates in cell culture, but requires additional seeding with preformed fibrils (PFFs) as was shown in FTDtau^1^-expressing rodent primary (Calafate et al., 2015; Wiersma et al., 2019) and hiPSC-derived neurons (Verheyen et al., 2015). In agreement, neurons expressing FTDtau^1^ in our co-culture system only developed tau aggregates upon seeding with PFFs of the K18 fragment of FTDtau^1^ at Week 2 (Supplementary Figure S3A). Confocal microscopy showed that seeding induces the formation of MC1-positive aggregates at Week 4 (Supplementary Figure S3B). Next, we used methanol (MeOH) fixation to remove soluble proteins (Guo et al., 2016; Wiersma et al., 2019) and subsequent AT100 immunolabelling confirmed that only in the presence of PFFs, FTDtau^1^ forms insoluble and pathologically phosphorylated aggregates (Supplementary Figure S3C). This demonstrates that neurons expressing FTDtau^1^ in our system develop intraneuronal tau aggregates when seeded by PFFs in a timeline comparable to that observed in rodent primary cells.

Exogenous seeds are intrinsically heterogeneous (Siddiqua et al., 2012) and can directly cause the degeneration of hiPSC-derived neurons (Usenovic et al., 2015). Moreover, tau seeds can trigger an inflammatory response in primary mouse (Wang and Ye, 2021) and human astrocytes (Ungerleider et al., 2021) and induce astrocyte-mediated synapse loss in a mouse neuron–astrocyte co-culture (Piacentini et al., 2017), making it a strong confounding factor in the study of cell (non-)autonomous responses to intraneuronal tau aggregation. Recently, expression of tau containing the FTD-associated P301L and S320F mutations was shown to enable seed-independent aggregation in rodent neurons *in vitro* (Strang et al., 2018), brain slice cultures (Croft et al., 2019) and *in vivo* (Koller et al., 2019). Therefore, we expressed fluorescently tagged human 2N4R tau with the P301L (FTDtau^1^) and S320F (FTDtau^2^) mutations either separately or combined (FTDtau^1+2^) selectively in neurons by viral transduction 1 day before astrocyte plating (Figure 1B and D). Neuronal expression of FTDtau^1+2^, but not FTDtau^1^ or FTDtau^2^, induced the formation of MC1-positive inclusions within 4 weeks of co-culture (Figure 1E), demonstrating intraneuronal aggregation of pathologically folded tau in the absence of seeds. MeOH fixation and subsequent AT8 and AT100 immunolabelling revealed that in contrast to FTDtau^1^, FTDtau^1+2^ spontaneously forms insoluble and pathologically phosphorylated tau aggregates (Supplementary Figure S4A and B). In addition, biochemical extraction and subsequent western blot analysis showed that expression of double mutant FTDtau^1+2^, but not single mutant FTDtau^1^ or FTDtau^2^, resulted in the formation of sarkosyl-insoluble tau aggregates (Supplementary Figure S4C). These data also showed that endogenous tau, distinguished by its lower molecular weight, does not co-aggregate with FTDtau^1+2^. Finally, MC1-positive FTDtau^1+2^ was positive for the thiophene dye HS169 (Figure 1F; Shirani et al., 2015), demonstrating that these inclusions consisted of stacked beta-sheets typically found in tau inclusions in the human brain. These data demonstrate seed-independent, intraneuronal tau aggregation in our human neuron–astrocyte co-culture model. Moreover, since aggregation only occurred in the presence of double mutant FTDtau^1+2^, overexpression of FTDtau^1^ provides an exquisite overexpression control to ascertain that effects in the co-culture are due to tau aggregation.

To further characterize and quantitatively assess the progression of tau pathology in our human co-culture model, we used automated, high-content microscopy. Since intraneuronal aggregation of FTDtau^1+2^ results in non-uniform fluorescence intensity (Figure 1E and F), tau aggregation was addressed using the fluorescence intensity standard deviation (SD) that has previously been used as a measure of tau aggregation in solution (Sasaki et al., 2019). Co-cultures expressing FTDtau^1+2^ were MeOH-fixed after 4 weeks and the fluorescence SD was compared with MC1 labelling within MAP2-positive neurons. This showed a strong positive correlation (*r* = 0.95) between the SD of FTDtau^1+2^ and labelling by MC1 (Supplementary Figure S4C), demonstrating the validity of this fluorescence measure to quantify intraneuronal tau aggregation.

Next, to assess the timing and specificity of FTDtau^1+2^ aggregation, we measured the fluorescence intensity and SD in untransduced, FTDtau^1^- and FTDtau^1+2^-expressing co-cultures at Weeks 1, 2.5, and 4 by high-content microscopy (Supplementary Figure S5A). These data confirm that, although FTDtau^1^ and FTDtau^1+2^ were expressed at similar levels (Supplementary Figure S5B and C), only FTDtau^1+2^ formed aggregates from 2.5 weeks, whereas aggregates were absent in both untransduced and FTDtau^1^-expressing co-cultures (Supplementary Figure S5D and E). These data demonstrate that tau expression and aggregation can be quantified by automated microscopy and further validate the use of FTDtau^1^ as control to determine whether effects of FTDtau^1+2^ in the co-culture are due to tau aggregation and not merely to the overexpression of mutant tau.

To address effects on neuronal development in FTDtau^1^- and FTDtau^1+2^-overexpressing co-cultures, we assessed the increase of morphological complexity at Weeks 1, 2, 3, and 4 by high-content microscopy (Supplementary Figures S1 and S6A). Dendrite segments, extremities, and branch points per neuron strongly increased over time with similar slopes for FTDtau^1^- and FTDtau^1+2^-expressing neurons (Supplementary Figure S6B–G), indicating that the formation of tau aggregates by FTDtau^1+2^ does not affect the development of hiPSC-derived neurons. In conclusion, by neuron-specific introduction of FTDtau^1+2^, we established and validated a novel human co-culture model of seed-independent, intraneuronal tau aggregation.

### Intraneuronal tau aggregation in human co-culture is progressive without immediate neurotoxicity

**Figure 2:**
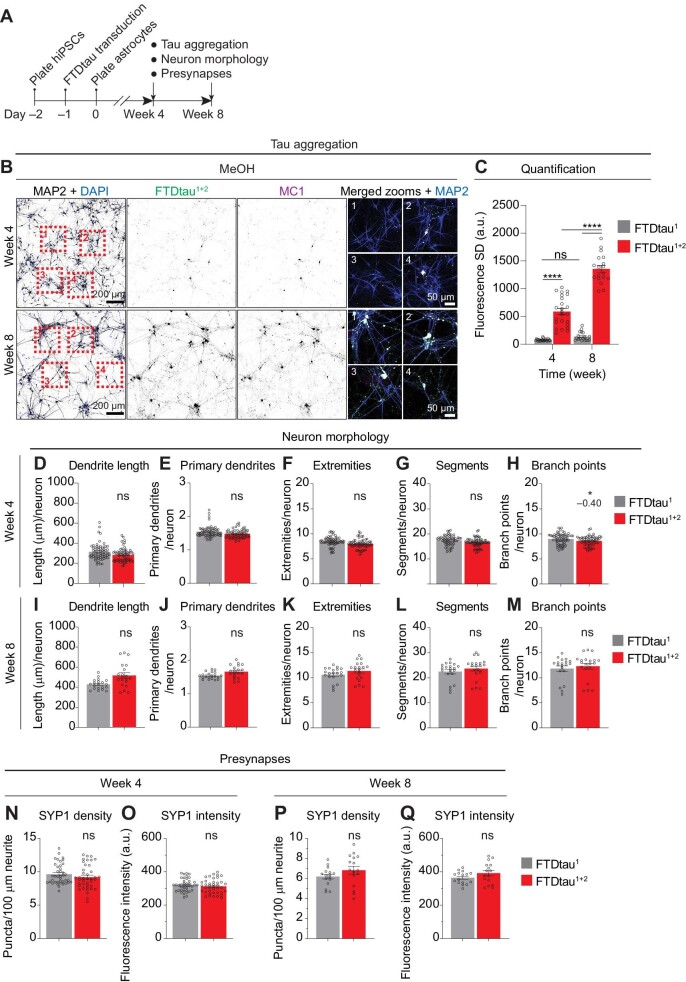
Intraneuronal tau aggregation is progressive without immediate neurotoxicity. (**A**) Schematic representation of the timeline of the experiment. Human neuron–astrocyte co-cultures were transduced with EGFP-tagged FTDtau^1^ or FTDtau^1+2^. At 4 and 8 weeks, soluble FTDtau was removed by MeOH fixation before immunostaining; neuron morphology and presynapses were quantified after PFA fixation by automated microscopy. (**B**) Representative confocal images show FTDtau^1+2^-EGFP direct fluorescence and immunostainings for MAP2 and pathological tau (MC1) in greyscale. Cell nuclei were visualized using DAPI (blue). Zooms of the numbered boxed areas, indicated in the MAP2 + DAPI panels, are shown as merged images of MAP2 (blue), FTDtau^1+2^ (green), and MC1 (magenta). (**C**) Quantification by automated microscopy of EGFP fluorescence SD in co-cultures transduced with EGFP-tagged FTDtau^1^ or FTDtau^1+2^. *n* = 3 independent experiments; *n* = 24 and 18 wells were analysed at 4 and 8 weeks, respectively. Bar graphs show mean ± SEM; data points represent mean/well. After Shapiro–Wilk normality testing, statistical analysis was performed by two-way ANOVA and Tukey's post-hoc analysis. ****P < 0.0001. ns, not significant. (**D**–**M**) Quantification of neuron morphology at Week 4 (**D**–**H**) and Week 8 (**I**–**M**) in co-cultures transduced with FTDtau^1^ and FTDtau^1+2^ and immunostained for MAP2 to assess dendrite length (**D** and **I**), primary dendrites (**E** and **J**), extremities (**F** and **K**), segments (**G** and **L**), and branch points (**H** and **M**) per neuron. *n* = 17 independent experiments and *n* = 64 wells were analysed for **D**–**H**; *n* = 4 independent experiments and *n* = 20 wells were analysed for **I**–**M**. (**N**–**Q**) Quantification of presynapses at Week 4 (**N** and **O**) and Week 8 (**P** and **Q**) in co-cultures transduced with FTDtau^1^ and FTDtau^1+2^ for presynapse (SYP1) density (**N** and **P**) and staining intensity (**O** and **Q**). *n* = 10 independent experiments and *n* = 40 wells were analysed at Week 4; *n* = 3 independent experiments and *n* = 16 wells were analysed at Week 8. Bar graphs show mean ± SEM; data points represent mean/well. For all datasets, statistical significance was assessed by Nested T test. *P < 0.05. ns, not significant. Supplementary Table S1 lists the total number of cells analysed and the exact *P*-values.

Since tau progressively accumulates in disease conditions, we investigated the temporal progression of intraneuronal tau pathology in our system. For this, we compared FTDtau^1+2^ aggregation at 4 and 8 weeks of co-culture using MeOH fixation and subsequent MC1 and MAP2 immunolabelling (Figure 2A). Confocal imaging demonstrated increased MeOH-insoluble, pathological tau aggregates in FTDtau^1+2^-expressing neurons upon prolonged co-culturing (Figure 2B). Automated microscopy and analysis confirmed increased insoluble FTDtau^1+2^ accumulation (∼2.3-fold) between Week 4 and Week 8 (Figure 2C), thus demonstrating progressive tau pathology in this co-culture system. Importantly, these data also demonstrate that also with prolonged culturing FTDtau^1^-expressing co-cultures do not spontaneously form tau aggregates.

Above we showed that the overexpression of mutant tau *per se* does not affect neuronal morphology, viability, and development (Supplementary Figures S2 and S6). However, in the human brain, the progression of tau pathology particularly correlates closely with the loss of presynapses (De Wilde et al., 2016). Therefore, we quantified the neuronal morphology and synapses in FTDtau^1^- and FTDtau^1+2^-expressing co-cultures at 4 and 8 weeks by high-content microscopy to assess the effect of progressive tau aggregation (Figure 2A; Supplementary Figure S1B, iii and iv). Because presynaptic SYP1 and postsynaptic PSD95 immunosignals overlap in our model (Figure 1A), the density and staining intensity of presynaptic SYP1 protein alone was used to assess effects on synapses. Since only a very slight reduction in branch points was observed at 4 weeks, but not at 8 weeks, we therefore concluded that neuron morphology (Figure 2D–M) and SYP1 density and intensity (Figure 2N–Q) were not different between FTDtau^1^- and FTDtau^1+2^-expressing co-cultures throughout the time course of the experiment. These data show that the morphology and synapses of neurons in our co-culture model are not affected by progressive tau aggregation, indicating that the model reflects an early disease stage prior to overt neurodegeneration. Moreover, comparison of FTDtau^1^- and FTDtau^1+2^-expressing co-cultures that both overexpress mutant tau, but differ in tau aggregation facilitates the study of the cellular effects of tau aggregation while controlling for overexpression of mutant tau.

### Intraneuronal tau aggregation progressively induces oxidative stress and activation of the ISR in astrocytes

**Figure 3:**
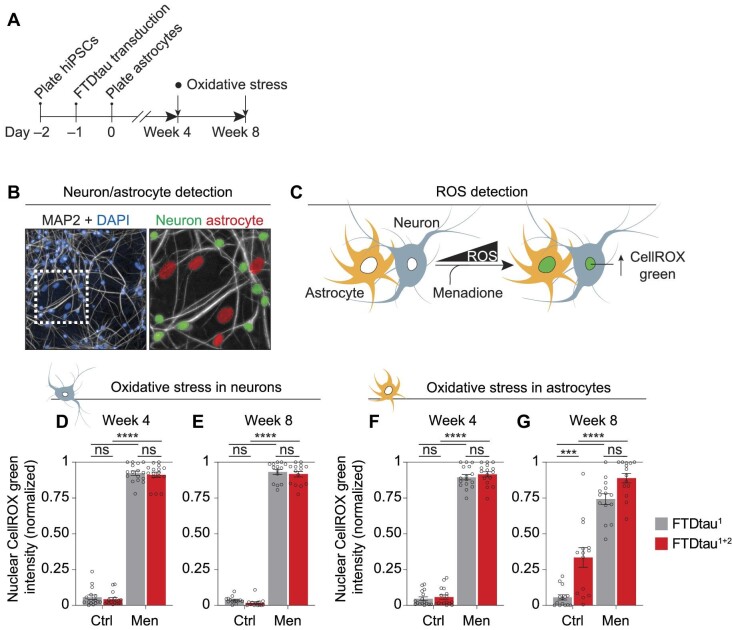
Intraneuronal tau aggregation progressively induces oxidative stress in astrocytes. (**A**–**C**) Human neuron–astrocyte co-cultures were transduced with mCherry-tagged FTDtau^1^ or FTDtau^1+2^ and oxidative stress was measured at 4 and 8 weeks in co-culture. (**A**) Schematic representation of the timeline of the experiment. (**B**) Representative widefield image captured by automated microscopy of a co-culture at 4 weeks show MAP2 immunostaining (grey) and nuclei visualized by DAPI (blue). The zoomed area, indicated in the MAP2 + DAPI channel, shows analysis using the automated protocol to distinguish MAP2-positive (green; neurons) and MAP2-negative (red; astrocytes) nuclei to quantify nuclear fluorescence of the ROS probe CellROX green and ISR target ATF4 in neurons and astrocytes. (**C**) Schematic representation of the experimental set-up to measure oxidative stress in neurons and astrocytes using CellROX green nuclear intensity in the absence or presence of Men. (**D**–**G**) Co-cultures were transduced with mCherry-tagged FTDtau^1^ or FTDtau^1+2^ and treated with 100 μM Men or vehicle control (Ctrl) for 3.5 h before loading with CellROX green. Immunostaining was performed for MAP2 and cell nuclei were visualized using DAPI. CellROX green intensities in neuronal/astrocytic nuclei at 4 (**D** and **F**) and 8 weeks (**E** and **G**) were quantified. *n* = 4 independent experiments; *n* = 16 and 14 wells were analysed at 4 and 8 weeks, respectively. Bar graphs show mean ± SEM and data points represent mean/well. The lowest and highest values per experiment were set to 0 and 1, respectively. After Shapiro–Wilk normality testing, statistical analysis was performed by two-way ANOVA and Tukey's post-hoc analysis. ***P = 0.001, ****P < 0.0001. ns, not significant. Widefield images of nuclear CellROX green fluorescence at 8 weeks are shown in Supplementary Figure S7A and B. Supplementary Table S1 lists the exact *P*-values and total number of cells analysed.

Oxidative stress is implicated as an early event of neurodegenerative tauopathies (Kamat et al., 2016) and was previously observed in mouse primary neurons with tau aggregation (Brelstaff et al., 2018). Since neurons greatly rely on astrocytes for antioxidant support (Bell et al., 2015), we assessed whether intraneuronal tau aggregation leads to an increase in reactive oxygen species (ROS) in neurons and astrocytes in co-culture (Figure 3A). Automated detection of neuronal and astrocytic nuclei allowed cell type-specific quantification of nuclear CellROX green, a fluorescent indicator of ROS in co-culture (Figure 3B and C). This showed that FTDtau^1^ or FTDtau^1+2^ expression did not induce ROS accumulation or sensitize for menadione (Men)-induced oxidative stress in neurons at 4 and 8 weeks in co-culture (Figure 3D and E; Supplementary Figure S7A). In contrast, ROS accumulation was observed in astrocytes in co-culture with FTDtau^1+2^-expressing neurons at 8 weeks, but not at 4 weeks (Figure 3F and G; Supplementary Figure S7B). At 8 weeks, neurons and astrocytes in FTDtau^1^-expressing co-cultures did not display increased ROS accumulation compared to untransduced cultures (Supplementary Figure S8A and B), indicating that the ROS accumulation in astrocytes in FTDtau^1+2^-overexpressing co-cultures is caused by intraneuronal tau aggregation and not due to overexpression of mutant tau. Altogether, these data demonstrate that astrocytes develop oxidative stress as a cell non-autonomous response to intraneuronal tau aggregation.

**Figure 4:**
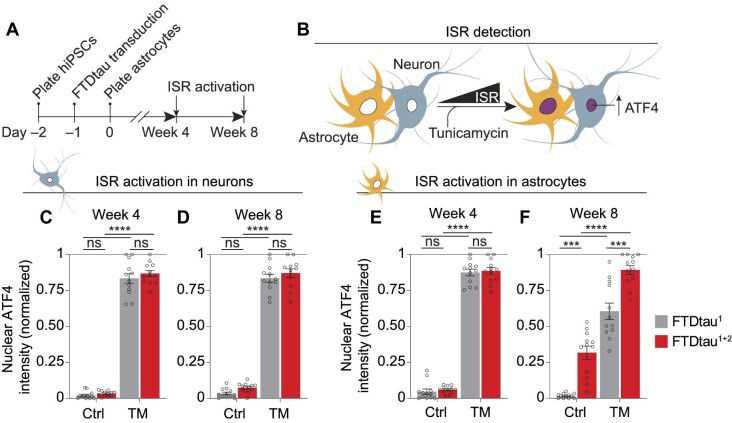
Intraneuronal tau aggregation induces the ISR in astrocytes. (**A** and **B**) Schematic representation of the timeline of the experiment (**A**) and experimental set-up (**B**). (**C**–**F**) Human neuron–astrocyte co-cultures were transduced with EGFP-tagged FTDtau^1^ or FTDtau^1+2^ and treated for 48 h with 10 μg/ml TM or vehicle control (Ctrl). Immunostaining was performed for MAP2 and ATF4, and cell nuclei were visualized using DAPI. ISR activation was determined by quantifying nuclear ATF4 intensity in neuronal and astrocytic nuclei by high-content microscopy (see Figure 3B) at 4 (**C** and **E**) and 8 (**D** and **F**) weeks. *n* = 3 independent experiments and *n* = 12 wells. Bar graphs show mean ± SEM and data points represent mean/well. The lowest and highest values per experiment were set to 0 and 1, respectively. Shapiro–Wilk normality testing followed by two-way ANOVA with Tukey's post-hoc analysis. ***P < 0.001, ****P < 0.0001. ns, not significant. Widefield images of nuclear ATF4 immunoreactivity at 8 weeks are shown in Supplementary Figure S7C and D. Supplementary Table S1 lists the exact *P*-values and total number of cells analysed.

Next, we assessed whether the ROS accumulation is accompanied by activation of the ISR. The ISR is a key proteostatic pathway that is induced during oxidative and other types of cellular stress and has been implicated in the pathogenesis of tauopathies (Scheper and Hoozemans, 2015; Grubman et al., 2019; Costa-Mattioli and Walter, 2020). To address ISR activation, we employed a validated high-content microscopy approach (Wolzak et al., 2022) to quantify cell type-specific nuclear immunopositivity for the ISR target ATF4 (Harding et al., 2000) via the automated distinction of neuronal and astrocytic nuclei (Figure 3B) in co-cultures at 4 and 8 weeks (Figure 4A and B). This showed that FTDtau^1^ or FTDtau^1+2^ expression did not induce ISR activation or sensitize for tunicamycin (TM)-induced ISR activation in neurons at 4 and 8 weeks (Figure 4C and D; Supplementary Figure S7C). In contrast, ISR activation and increased sensitivity for TM-induced ISR activation was observed in astrocytes in co-culture with FTDtau^1+2^-expressing neurons at 8 weeks, but not at 4 weeks (Figure 4E and F; Supplementary Figure S7D). ISR activation was not observed in neurons or astrocytes in FTDtau^1^-expressing co-cultures compared to untransduced cultures (Supplementary Figure S8C and D). This excludes that the ISR activation in astrocytes in FTDtau^1+2^-expressing co-cultures is an effect of overexpression of mutant tau. Altogether, these data demonstrate that intraneuronal tau aggregation prior to neurodegeneration induces oxidative stress and activation of the ISR specifically in astrocytes.

Finally, we investigated whether the cell non-autonomous induction of oxidative stress and the ISR in astrocytes requires direct contact with neurons. To this end, monocultured human astrocytes were exposed to conditioned media of 8-week-old co-cultures that were untransduced or expressing FTDtau^1^ or FTDtau^1+2^ (Supplementary Figure S9A). Quantification of nuclear CellROX green and ATF4 immunoreactivity by automated high-content microscopy showed increased ROS accumulation and ISR activation upon Men and TM treatment, respectively (Supplementary Figure S9B–E), confirming that monocultured astrocytes have intact stress responses. However, no differences in ROS accumulation and ISR activation were observed in monocultured astrocytes exposed to conditioned media of untransduced, FTDtau^1^- and FTDtau^1+2^-expressing co-cultures (Supplementary Figure S9F–I), indicating that the cell non-autonomous responses of astrocytes to intraneuronal tau aggregates that are present in direct co-culture do not readily transfer via conditioned media. In conclusion, these data show that intraneuronal tau aggregates induce oxidative stress and ISR activation in astrocytes through a mechanism that requires proximity to and/or direct contact with tau aggregate-containing neurons.

### Antisense *targeting of intraneuronal tau aggregates prevents oxidative stress and ISR activation in astrocytes*

To further investigate the connection between tau aggregation in neurons and cellular stress responses in astrocytes, we employed MAPT (the gene encoding tau) antisense oligonucleotides (AONs) to interfere in tau aggregation. MAPT AONs that lower tau mRNA and protein as well as extracellular tau levels were previously shown to reduce tau aggregation and halt disease progression in transgenic mice (DeVos et al., 2017; Mignon et al., 2018). Since the intracellular delivery of unmodified AONs is generally inefficient, we employed a 3′ cholesteryl-conjugate attached via a triethylene glycol linker and tested the effect of a lipid carrier (lipofection). Using a 5′ Cy3 fluorescent marker to visualize intracellular delivery, untransduced co-cultures were treated at Week 2 with 100 nM modified MAPT AON in the absence or presence of lipofectamine, and Cy3 fluorescence was quantified in MAP2-positive neurons at Week 4 (Supplementary Figure S10A). Confocal microscopy showed that, although 3′ cholesteryl AON is detected in neurons without lipofection, AON delivery was strongly facilitated by the presence of lipofectamine (Supplementary Figure S10B). This was confirmed by quantification with automated microscopy (Supplementary Figure S10C), demonstrating that efficient delivery of MAPT AON in human neuron–astrocyte co-culture requires lipofection. Because conjugation may interfere with AON activity (Schlagnitweit et al., 2019) we continued with unmodified AONs combined with lipofectamine and optimized the dose in untransduced co-cultures (Supplementary Figure S10D). Widefield microscopy showed excessive clustering of Tuj1-positive neurons only at higher concentration (500 nM) of scramble and MAPT AON treatment (Supplementary Figure S10E), indicative of lipofectamine toxicity, and 100 nM AON was selected for targeting tau in subsequent experiments.

First, AON target engagement was tested by treating FTDtau^1^- and FTDtau^1+2^-expressing co-cultures with 100 nM MAPT or scramble AONs preceding tau expression and aggregation at Week 1 and Week 2 (Supplementary Figure S10F). Confocal microscopy showed that MAPT AON treatment strongly reduced the total levels of FTDtau^1+2^ (Supplementary Figure S10G). In addition, MAPT AON treatment prevented the formation of MC1-positive FTDtau^1+2^ inclusions. Quantification by automated microscopy confirmed strongly reduced total levels of FTDtau^1^ (–64%) and FTDtau^1+2^ (–74%) in neurons upon MAPT AON treatment compared to scramble AON treatment (Supplementary Figure S10H). Moreover, fixation and subsequent MC1 immunolabelling demonstrated that MAPT AON treatment prevented the formation of insoluble, MC1-positive FTDtau^1+2^ at 4 weeks (Supplementary Figure S10I). Quantification by automated microscopy confirmed that, as expected, no tau aggregation occurred for FTDtau^1^ in the presence of scramble or MAPT AONs (Supplementary Figure S10J). Interestingly, the aggregation of FTDtau^1+2^ was prevented by treatment with MAPT AON, but not scramble AON. Altogether, these data show that antisense therapy can efficiently target tau aggregation in our human co-culture system.

**Figure 5:**
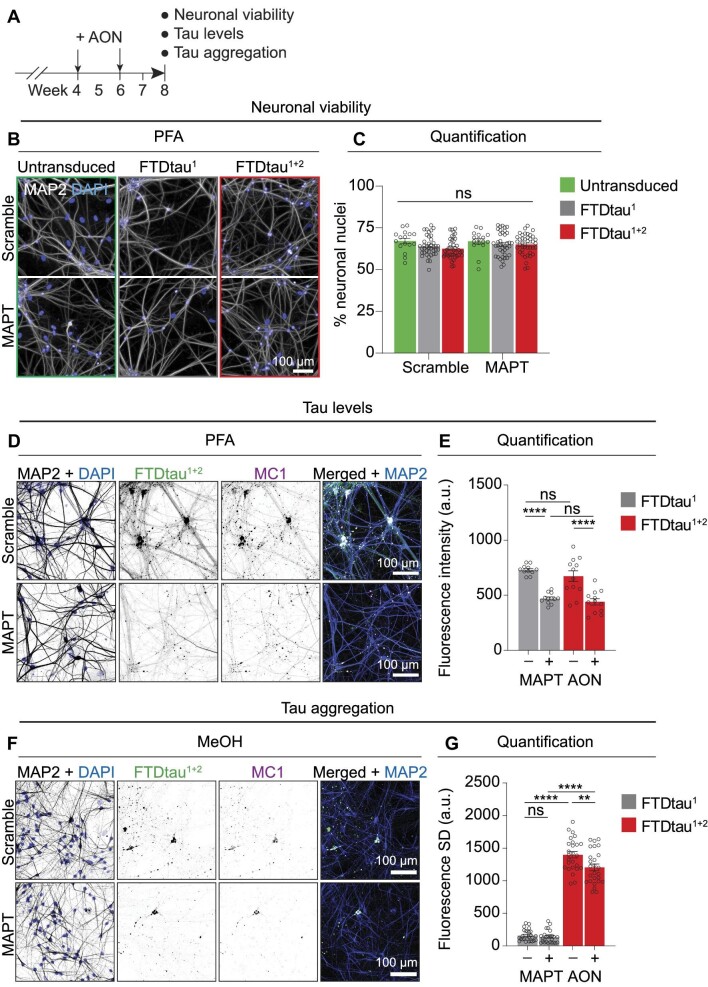
MAPT antisense therapy reduces tau aggregation in human co-culture. (**A**) Schematic outline of the experiment. Co-cultures were treated at Week 4 and Week 6 with MAPT or scramble AONs. Neuronal viability as well as tau expression and aggregation was assessed at 8 weeks. See Materials and methods for more detail. (**B** and **C**) Co-cultures without or with transduction with EGFP-tagged FTDtau^1^ or FTDtau^1+2^ were treated with scramble or MAPT AONs in the presence of 0.5 μl lipofectamine per μg of AON. (**B**) Representative widefield images captured by automated microscopy of PFA-fixed co-cultures at 8 weeks show MAP2 immunostaining (grey) and nuclei visualized by DAPI (blue). (**C**) Neuronal viability was determined by the percentage of neurons in co-cultures at 8 weeks. (**D** and **F**) Representative confocal images at 8 weeks of total (PFA-fixed, **D**) and insoluble (MeOH-fixed, **F**) EGFP-tagged FTDtau^1+2^ in neuron–astrocyte co-cultures, treated with scramble (–) or MAPT (+) AONs. EGFP direct fluorescence and immunostainings for MAP2 and a pathological conformation of tau (MC1) are shown in greyscale. Cell nuclei were visualized using DAPI (blue). Merged images include MAP2 (blue), FTDtau^1+2^ (green), and MC1 (magenta). (**E** and **G**) Quantification by automated microscopy of EGFP mean fluorescence intensity from **D** showing total tau levels and EGFP mean fluorescence SD from **F** showing aggregated tau levels, respectively, in co-cultures. Bar graphs in **C, E**, and **G** show mean ± SEM, and data points represent mean/well. For **C**, *n* ≥ 4 independent experiments, *n* ≥ 16 wells; for **E**, *n* = 2 independent experiments, *n* ≥ 11 wells; for **G**, *n* = 4 independent experiments, *n* = 26 wells. Shapiro–Wilk normality testing followed by two-way ANOVA with Tukey's post-hoc analysis. **P < 0.01, ****P < 0.0001. ns, not significant. Supplementary Table S1 lists the exact *P*-values and total number of cells analysed.

To enhance translational relevance, co-cultures were treated after the onset of tau aggregation with MAPT and scramble AONs at Week 4 and Week 6 and analysed at Week 8 (Figure 5A). Automated microscopy showed no difference in neuronal viability between untransduced and FTDtau^1^- or FTDtau^1+2^-expressing co-cultures (Figure 5B and C). This demonstrates that, similar to 4-week-old co-cultures, neither expression of FTDtau variants nor the tau aggregation that is only present in FTDtau^1+2^-expressing co-cultures affect neuronal viability at 8 weeks of co-culture. Also, no differences in neuronal viability were found in the co-cultures treated with scramble or MAPT AON (Figure 5B and C). This indicates that neurons, irrespective of their tau load, are not negatively affected by AON therapy in this human co-culture model. Importantly, confocal microscopy showed that compared to scramble, MAPT AON treatment reduced the total levels of FTDtau^1+2^ (Figure 5D). In addition, MAPT AON treatment reduced the formation of MC1-positive FTDtau^1+2^ inclusions. This was confirmed and quantified by automated microscopy, showing that neurons in co-cultures that were treated with MAPT AON presented with significantly reduced levels of FTDtau^1^ (–32%) and FTDtau^1+2^ (–34%) compared to scramble conditions at Week 8 (Figure 5E). Moreover, MC1 immunolabelling after MeOH fixation showed that MAPT AON treatment reduced insoluble, MC1-positive FTDtau^1+2^ (Figure 5F). This was confirmed by automated microscopy, showing a significant reduction of FTDtau^1+2^ aggregation (–14%) compared to scramble conditions at Week 8 (Figure 5G). Therefore, MAPT AON treatment after the onset of tau aggregation reduces the progressive accumulation of insoluble tau aggregates in our human co-culture model.

To assess the effect of antisense treatment on extracellular tau in our co-culture model, we measured total tau levels in the conditioned medium of 8-week-old untransduced and FTDtau^1^- and FTDtau^1+2^-expressing co-cultures, in the absence and presence of scramble or MAPT AON treatment at Week 4 and Week 6. These data showed the presence of tau in conditioned media of untransduced co-cultures (Supplementary Figure S11), demonstrating the release of tau in our co-culture model in agreement with previous observations (Pooler et al., 2013). Increased extracellular tau levels were observed in conditioned media of FTDtau^1^-expressing cultures. The extracellular tau levels in FTDtau^1+2^-expressing cultures were not different from untransduced cultures. Importantly, there was no difference in extracellular tau levels between FTDtau^1^- and FTDtau^1+2^-expressing co-cultures. Moreover, irrespective of FTDtau expression, scramble and MAPT AON treatment did not affect extracellular tau levels, demonstrating that the reduction of intracellular tau levels and aggregation by antisense therapy described earlier (Figure 5D–G) are not reflected in extracellular tau levels during the time course of the experiment. Altogether, these data demonstrate that extracellular tau levels are not different between FTDtau^1^- and FTDtau^1+2^-expressing co-cultures and are not affected by antisense therapy in our model. These data suggest that the cell non-autonomous astrocytic stress responses to intraneuronal tau aggregates not induced by altered extracellular tau levels *per se.*

**Figure 6:**
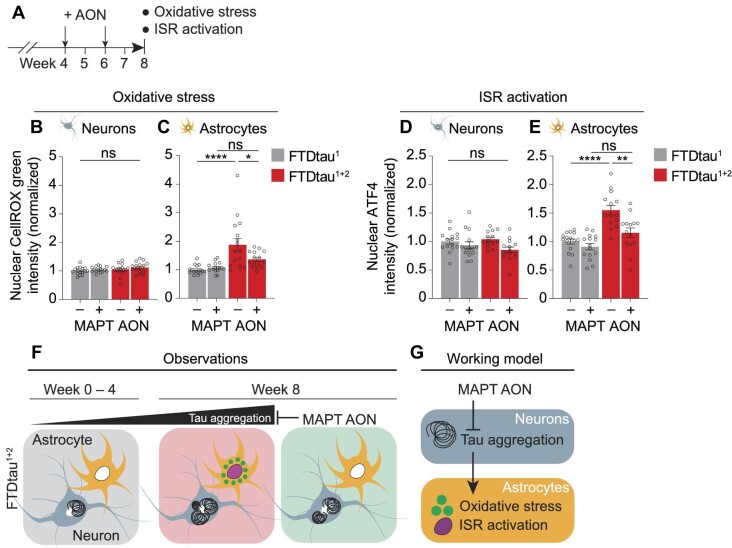
MAPT antisense therapy prevents tau-mediated oxidative stress and ISR activation in astrocytes in human co-culture. (**A**) Schematic outline of the experiment. (**B**–**E**) Co-cultures transduced with mCherry- (**B** and **C**) or EGFP-tagged (**D** and **E**) FTDtau^1^ or FTDtau^1+2^ were treated with scramble (–) or MAPT (+) AONs at Week 4 and Week 6. CellROX green (ROS) or ATF4 (ISR) nuclear intensity was quantified by high-content microscopy in neurons and astrocytes in co-cultures. Bar graphs show mean ± SEM and data points represent the fold-change difference per well over scramble treatment in FTDtau^1^-expressing co-cultures for each independent experiment. *n* = 4 independent experiments and *n* ≥ 14 wells were analysed. Shapiro–Wilk normality testing followed by two-way ANOVA with Tukey's post-hoc analysis. *P < 0.05, **P < 0.01, ****P < 0.0001. ns, not significant. Supplementary Table S1 lists the exact *P*-values and total number of cells analysed. (**F**) Schematic overview of the key observations in the human co-culture model of tau aggregation. (**G**) The working model for tau-mediated cell non-autonomous cellular stress. Tau antisense therapy prevents oxidative stress and activation of the ISR in astrocytes by reducing intraneuronal tau aggregation.

Finally, we tested whether MAPT AON treatment could ameliorate the oxidative stress and ISR activation in astrocytes in response to intraneuronal tau pathology. To this end, FTDtau^1^- and FTDtau^1+2^-expressing co-cultures were treated with MAPT or scramble AONs at Week 4 and Week 6, and nuclear fluorescence intensity of the ROS probe CellROX green or the ISR target ATF4 were determined in neurons and astrocytes by automated microscopy at Week 8 (Figure 6A). Indeed, with scramble AON treatment, increased ROS accumulation and ATF4 in astrocytes was observed in astrocytes in FTDtau^1+2^- but not FTDtau^1^-expressing co-cultures (Figure 6B–E), thus corroborating our previous observation that intraneuronal tau aggregation rather than expression of mutant tau induces oxidative stress and ISR activation in astrocytes. Importantly, MAPT AON treatment prevented the increase in ROS levels in astrocytes in co-cultures with FTDtau^1+2^ aggregation (Figure 6B and C). Moreover, MAPT AON treatment also blocked the increased nuclear ATF4 levels in astrocytes in co-cultures with intraneuronal tau aggregation induced by FTDtau^1+2^ (Figure 6D and E). Together, these data show that cell non-autonomous oxidative stress and ISR activation in astrocytes is an effect of intracellular tau aggregation in neurons that can be prevented by tau-targeted antisense therapy.

## Discussion

Here we developed a miniaturized human neuron–astrocyte co-culture system with seed-independent, intraneuronal tau aggregation. We have established and extensively validated a quantitative automated, high-content microscopy methodology to study disease mechanisms in this robust and controlled model for tau aggregation. We show that progressive, intraneuronal tau aggregation induces oxidative stress and ISR activation in astrocytes (Figure 6F) indicating a cell non-autonomous stress response in astrocytes that can be prevented by tau-directed AON therapy (Figure 6G). Using tau-directed AONs, we are the first to demonstrate therapeutic tau intervention in a medium throughput, fully human, translationally relevant model of tau pathology, giving a unique opportunity for future target identification and intervention studies.

Previous tau aggregation models made use of a single FTD mutation, which after a few months results in spontaneous tau aggregation in mice (Denk and Wade-Martins, 2009). For efficient modelling of tau pathology in cell culture, the aggregation of overexpressed tau is triggered by the addition of preformed tau aggregates that function as a seed that initiates intracellular aggregation after internalization (Verheyen et al., 2015; Wiersma et al., 2019). Seeding also induces tau aggregation in our system, thus enabling the study of disease mechanisms pertaining to seeded tau aggregation in hiPSC-derived neurons. Yet, the relatively high level of extracellular tau aggregates is a potential confounding factor in the study of cell non-autonomous responses. In fact, the internalization of seeds by astrocytes has been shown to elicit oxidative stress, inflammatory and synaptotoxic responses (Piacentini et al., 2017; Ungerleider et al., 2021; Wang and Ye, 2021).

In this study, we focused on the effects of intraneuronal tau aggregation, as the most prominent pathological hallmark that correlates with neurodegeneration and cognitive decline. To obtain tau aggregation we overexpressed 2N4R tau with two FTD mutations. The combination of two pathogenic MAPT mutations has not been reported in patients, but the vastly increased spontaneous aggregation rate of FTDtau^1+2^ allows the modelling of tau aggregation without the use of seeds (Strang et al., 2018; Croft et al., 2019; Koller et al., 2019). We confirmed that FTDtau^1+2^ pathology in our model bears the characteristics of tau pathology in the human brain: it is positive for the commonly used antibodies AT8, AT100, and MC1, showing that the tau in our model is pathologically phosphorylated and adopts a pathological conformation. Moreover, the MeOH- and sarkosyl-insolubility as well as the labelling by the beta-sheet-binding dye HS169 demonstrate the formation of aggregates. Within the timeline of our experiments, the occurrence of tau pathology is dependent on the presence of two FTD mutations and is not observed with tau containing a single FTD mutation. Moreover, our data indicate that overexpression of FTDtau^1^ does not affect neuronal viability, morphology, and synapses, and does not induce oxidative stress or ISR activation compared to untransduced co-cultures. Hence, comparison of double mutant FTDtau^1+2^ with single mutant FTDtau^1^ provides an elegant control to ascertain that observed effects are due to tau aggregation rather than overexpression of mutant tau.

Tau pathology is connected to neurodegeneration in a heterogeneous group of diseases (Lee et al., 2001; Spillantini and Goedert, 2013). Recently it has become clear that tau aggregates in different tauopathies have different structures that are dictated by tau isoform, the presence of mutations and unknown factors (Frost et al., 2009; Kaufman et al., 2016; Fitzpatrick et al., 2017). These different tau strains may account for spatiotemporal spreading patterns in the human brain (Kaufman et al., 2016), but it is likely that they at least partly share a pathomechanism. The current model flexibly allows for implementation of different tau isoforms and mutations if required for specific research questions.

In this study, we have developed a 96-well co-culture system that is compatible with automated microscopy and analysis to quantify the timing, extent, and cell (non-) autonomous effects of intraneuronal tau aggregation. This platform enabled the quantitative assessment of morphological, synaptic, and cellular stress readouts in a standardized manner across multiple independent experiments, encompassing more than thousands of cells (see Supplementary Table S1). Our data evaluating antisense therapy indicates that this model can be used as a preclinical tool to screen tau-targeting therapeutics in a high-throughput manner. To enable the use of human neurons for this approach, we circumvented the long differentiation time and heterogeneity that is commonly associated with hiPSC-derived neurons (Volpato and Webber, 2020), by utilizing a clonal Ngn2 hiPSC-line for rapid induction of predominantly excitatory neurons (Frega et al., 2017). Excitatory neurons were previously shown preferentially vulnerable for tau accumulation (Fu et al., 2019) and subsequent degeneration (Fu et al., 2017). In future studies, GABAergic neurons may be incorporated to generate a balanced network (Kuijlaars et al., 2016), to model the early disruptions in network activity that have been observed in tauopathy mice (Stancu et al., 2015; Fu et al., 2017).

Rodent astrocytes support the differentiation of hiPSCs into functional neurons (Tang et al., 2013; Lischka et al., 2018) but this results in a mixed species co-culture. Alternatively, human astrocytes can be generated from hiPSCs and have been shown to support the maturation of hiPSC-derived neurons in co-culture (Hedegaard et al., 2020). However, conflicting results have been reported that can be explained by their relatively immature state compared to primary human astrocytes (Lischka et al., 2018). Therefore, to circumvent the heterogeneity in our microwell co-cultures that may arise due to the inefficient differentiation of hiPSC-derived astrocytes, we used commercially available primary human cortical astrocytes that were previously shown to support hiPSC-derived neurons in a similar format (Kuijlaars et al., 2016). In future studies, patient hiPSC-derived astrocytes may be implemented to study the effect of disease variants on the tau-mediated cell non-autonomous stress response.

To express FTDtau specifically in morphologically mature neurons in a co-culture with primary human astrocytes, we developed a co-culture protocol that is compatible with the viral transduction of Ngn2-neural progenitor cells. To exclude that expression of FTDtau variants interfered with neuronal maturation, we extensively validated this protocol and found no effects of viral transduction, tau overexpression, or aggregation on the morphological development, synapses, and viability of hiPSC-derived neurons, although we cannot fully exclude that subtle effects in a minor portion of neurons are not resolved by our population-level analysis. It is possible that the accelerated tau aggregation of the FTDtau^1+2^ variant precludes the detection of tau-associated changes in neurons that may take years to develop in the human brain. Moreover, neuron and synapse loss may require the presence of microglia. Studies in mice indicate that during the neuroinflammatory conditions of tauopathy, microglia and astrocytes collaborate in neuro- and synaptotoxic events via the release of complement factors and cytokines (Wu et al., 2019). Interestingly, in neuron–astrocyte–microglia tri-culture models for beta amyloid formation, human astrocytes and microglia produce such molecules (Guttikonda et al., 2021). It will therefore be interesting to incorporate human microglia in our current model to investigate the downstream effects of tau pathology on cytokine secretion, neuron–glia crosstalk, neuronal viability, and synapses.

Cell non-autonomous effects of neuronal tau pathology on astrocytic Notch signalling (Hasel et al., 2017) and the neuron-supportive function of astrocytes (Sidoryk-Wegrzynowicz et al., 2017) have previously been reported in mouse models. Recently, single-cell RNA profiling studies demonstrated that the oxidative stress/Nrf2 and ISR/ATF4 pathways are key transcriptional programs activated in astrocytes in tau transgenic mice (Jiwaji et al., 2022) and human AD entorhinal cortex (Grubman et al., 2019), respectively. These observations are in line with the results we report here and strongly support the translational value of our model. However, it is impossible to infer a causal sequence of events from the observational data obtained in brain tissue. In addition, there are additional potential confounding factors in this more complex system that may contribute to the phenotype. Our reduced human model allowed for the identification of a novel disease mechanism, showing a direct causal relation between intraneuronal tau aggregation and presence of oxidative stress as well as activation of the ISR in astrocytes. In addition, we show that this is an early pathogenic response that does not require the presence of microglia or other cell types.

The ISR is the downstream convergence point for the four stress-induced eIF2α kinases, that can be directly and indirectly activated by ROS (Costa-Mattioli and Walter, 2020). The resulting phosphorylation of the translation factor eIF2α reduces global translation and enhances the translation of specific mRNAs including that encoding the transcription factor ATF4 (Harding et al., 2000). Thus, phosphorylated eIF2α activates an ISR transcriptional program via ATF4. In addition, it can also induce a neuroinflammatory response through NFκB-mediated transcription (Sprenkle et al., 2017; Costa-Mattioli and Walter, 2020). In this respect it is interesting to note that activation of the astrocytic ATF4 pathway in human AD coincides with a p65–NFκB-mediated pro-inflammatory profile (Grubman et al., 2019). Modulation of the ISR is extensively investigated as a therapeutic strategy for the treatment of neurodegenerative disease (Costa-Mattioli and Walter, 2020). For example, genetic reduction and pharmacological inhibition of the eIF2α kinase PERK (Moreno et al., 2013; Radford et al., 2015; Scheper and Hoozemans, 2015), overexpression of the eIF2α phosphatase GADD34 (Moreno et al., 2012), or promoting its interaction with eif2B (Halliday et al., 2015) has been shown to be effective in mouse models for different neurodegenerative diseases including tauopathies. These studies showed exciting proof of concept in the context of the whole mouse brain, but the cell type that is targeted by these approaches is unknown. The relevance and therapeutic potential of the astrocytic ISR in particular is illustrated by a recent report showing that astrocyte-specific ISR intervention ameliorates disease in a mouse model for prion-mediated neurodegeneration (Smith et al., 2020).

The astrocytic response is observed in the absence of neuronal and synaptic loss and therefore not a non-specific response to dead cells or cellular debris. Our model therefore uniquely enables further dissection of the cell non-autonomous astrocytic contribution to tau pathology prior to neurodegeneration. In addition, our model will also provide an opportunity to gain mechanistic understanding of the pathways by which neuronal tau aggregation affects astrocytes. Previous studies have demonstrated that tau can be released from neurons (Pooler et al., 2013), possibly triggering an astrocytic response to extracellular tau upon internalization (Piacentini et al., 2017; Ungerleider et al., 2021; Wang and Ye, 2021). Like in the human brain (Ben Haim and Rowitch, 2016), not all astrocytes were detected by canonical immunomarkers in our co-culture. Therefore, despite the restricted tau expression and aggregation in neurons in our co-culture, low levels of intracellular tau in astrocytes cannot be fully excluded. Our data, however, demonstrate that although extracellular tau is present in the co-culture media, levels are similar between FTDtau^1^ and FTDtau^1+2^ and are therefore unlikely to drive the cell non-autonomous disease mechanisms of tau aggregation that are specific for the latter. This is further corroborated by our observation that tau-directed antisense therapy did not affect extracellular tau levels, yet prevented the stress response in astrocytes. Our model therefore indicates that intraneuronal tau aggregation does not induce the cell non-autonomous response in astrocytes via extracellular tau, although the involvement of a specific low-abundant and/or vesicle-contained extracellular tau species that may not have been detected by our bulk tau analysis cannot be excluded. Our data demonstrate that the cell non-autonomous stress responses are not transferred via conditioned media. This indicates that the tau-induced astrocytic response requires close proximity to and/or direct contact with neurons. This could involve the direct transfer of factors, as has been suggested for transfer of tau from neurons to astrocytes (Fleeman and Proctor, 2021; Maté De Gérando et al., 2021), for example via tunnelling nanotubes (Chastagner et al., 2020). Alternatively, the astrocytic response may depend on a local gradient of a diffusible factor or factors at the cell surface that becomes diluted in conditioned media. In addition, labile factor(s) may be involved that are not stable in conditioned media.

The tau-targeting AON used in this study was previously shown to halt disease progression and astrocyte reactivity in tauopathy mice by reducing tau expression (DeVos et al., 2017). Moreover, it was found effective and well-tolerated in nonhuman primates and has proceeded into clinical trial (NCT03186989) (Mignon et al., 2018). The acute knockdown of endogenous tau in mice by adeno-associated virus delivery of short-hairpin RNA (Velazquez et al., 2018) but not with zinc finger protein transcription factors (Wegmann et al., 2021) resulted in synaptic and behavioral deficits. Therefore, the safety and efficacy of tau-directed therapeutics need to be critically evaluated in a system without endogenous mouse tau that escapes targeting by the AON. We do not observe adverse effects of AON therapy on the viability of hiPSC-derived neurons, in line a previous study on the CRISPR/Cas9-mediated genetic knockdown of endogenous tau (Silva et al., 2016). We show that both in untransduced as well as FTDtau variant-overexpressing hiPSC-derived neurons in the same endogenous tau background, the acute AON-mediated reduction of tau is well-tolerated by human neurons.

Four weeks of AON treatment in human co-culture resulted in reduction, but not the reversal of tau aggregation that was previously observed in mice (DeVos et al., 2017). Despite the incomplete reduction of tau levels and the relatively mild effect on aggregation, AON treatment has a profound effect on astrocytes. It is possible that rather than the insoluble tau pool, reducing the levels of soluble aggregation intermediates is protective. This is in line with a previous study demonstrating that 6 weeks suppression of tau transgene expression rescues the neurodegenerative phenotype in a mouse model despite the continued presence of aggregates (Santacruz et al., 2005). In a similar mouse model, prolonged suppression of tau expression reversed tangle pathology (Polydoro et al., 2013). Together with the observations of reversed tau pathology by AON therapy in mice (DeVos et al., 2017) and the turnover of FTDtau^1+2^ aggregates with soluble FTDtau^1+2^ (Croft et al., 2021), this suggests that lowering soluble tau may shift an equilibrium that ultimately favors the disassembly of pre-existing aggregates. This indicates that suppressing tau expression by AON therapy may be a viable intervention strategy even at advanced stages of tauopathy.

Concluding, in the present study we established and extensively validated the first fully human neuron–astrocyte co-culture displaying seed-independent, progressive intraneuronal tau aggregation. Using this model, we identified oxidative stress and ISR activation in astrocytes as a cell non-autonomous response to intraneuronal tau aggregation. Moreover, we provide the first evidence for the effectivity of tau-directed AON therapy in a fully human model to reduce intraneuronal tau aggregation, as well as to prevent the cell non-autonomous response in astrocytes. This novel human model will provide new opportunities to further investigate disease mechanisms in neurons and astrocytes, and evaluate tau-directed therapeutics in a translationally relevant *in vitro* context.

## Materials and methods

### Stem cell and primary human astrocyte cultures

A previously generated hiPSC line stably expressing a doxycyclin-inducible *rtTA/Ngn2* (Frega et al., 2017) was maintained feeder-free on vitronectin (Stem Cell Technologies)-coated plates in TeSR-E8 medium containing 1× TeSR-E8 supplement (Stem Cell Technologies) and 50 U/ml Pen/strep (Gibco) supplemented with G418 (50 μg/ml; Sigma) and puromycin (0.5 μg/ml; Sigma) at 37°C/5% CO_2_ under hypoxic conditions. Colonies were fed daily and double-volume feeding allowed for weekend-free culturing. Once or twice a week, colonies were passaged by non-enzymatic dissociation with Gentle Cell Dissociation Reagent (Stem Cell Technologies) and replated in the presence of Rho-associated, coiled-coil-containing protein kinase inhibitor (RI; 10 μM; Selleckchem) for 24 h to promote cell survival. To prevent genomic instability and senescence by prolonged passaging of hiPSCs, cryopreserved cells were thawed 2 weeks prior to use once cells reached passage number 25.

Primary human foetal astrocytes (ScienCell #1800) were cultured in Astrocyte Medium (ScienCell; 1× Growth Supplement, 1× fetal bovine serum (FBS), and 100 U/ml Pen/strep) on Geltrex (1:100 in DMEM/F12)-coated plates and half of the medium was refreshed twice a week. Astrocytes were passaged once a week by enzymatic dissociation with Accutase (Sigma). Cultures were kept at 37°C/5% CO_2_ at physiological oxygen conditions and used until passage number 7. To eliminate variability in astrocyte physiology due to genetic background of the donor, all experiments in this study were conducted with cryopreserved cells that were expanded from one vial.

### Human neuron–astrocyte co-culture

On Day –2 (i.e. 2 days prior to astrocyte addition), hiPSCs were non-enzymatically dissociated with Gentle Cell Dissociation Reagent and plated as single cells (4000 cells/well) in black 96-well culture plates (Greiner #655090) precoated with Geltrex (1:100 in DMEM/F12) in TesR-E8 medium with NT3 (10 ng/ml; Peprotech), BDNF (10 ng/ml; Peprotech), RI (10 μM), and doxycycline (2 μg/ml; Sigma) for induction of the Ngn2 transgene. One day after plating, medium was changed to DMEM/F12 (Gibco; 1× N2 supplement, 1× NEAA, 100 U/ml Pen/strep) with NT3 (10 ng/ml), BDNF (10 ng/ml), and doxycycline (2 μg/ml). At Day 0, primary human astrocytes were enzymatically dissociated with Accutase and plated as single cells (4000 cells/well) in Neurobasal Medium (Gibco; 1× B27 + VitA, 1× Glutamax, and 100 U/ml Pen/strep) with NT3 (10 ng/ml), BDNF (10 ng/ml), and doxycyclin (2 μg/ml) on top of Ngn2-neural progenitors to enhance neuronal maturation. One day after astrocyte plating (i.e. Day 1), half of the medium was refreshed with Neurobasal Medium containing NT3 (10 ng/ml), BDNF (10 ng/ml), doxycyclin (2 μg/ml), and 1-β-D-arabinofuranosylcytosine (4 μM; Millipore) to stop proliferation of dividing cells. At Day 2 (i.e. 2 days after astrocyte plating), half of the medium was refreshed with medium containing only NT3 (10 ng/ml) and BDNF (10 ng/ml). Starting from 1 week after astrocytes plating, half of the medium was refreshed once a week and supplemented with 0.5% FBS (Gibco) to support astrocyte viability. Two days prior to MeOH fixation, Geltrex (1:40 in complete medium) was added to the co-culture medium (1:200 final dilution) to prevent dissociation of co-cultures. Only the inner 32 wells of the 96-well plate were used for culturing and empty wells were filled with sterile phosphate-buffered saline (PBS) to minimize evaporation of the culture medium. Throughout the neuronal differentiation process, co-cultures were kept at 37°C/5% CO_2_ at physiological oxygen conditions.

### Astrocyte monocultures in conditioned media

Conditioned media were removed from neuron–astrocyte co-culture after 8 weeks and centrifuged at 2000 relative centrifugal force (rcf) for 3 min and frozen at –20°C until use. Primary human astrocytes were enzymatically dissociated with Accutase and plated as single cells (4000 cells/well) in black 96-well culture plates (Greiner #655090) precoated with Geltrex (1:100 in DMEM/F12) in Neurobasal Medium (Gibco; 1× B27 + VitA, 1× Glutamax, 100 U/ml Pen/strep, and 0.5% FBS) with NT3 (10 ng/ml) and BDNF (10 ng/ml). One day after plating, medium was replaced by conditioned media and cultures were kept for 1 week until fixation.

### Lentiviral transduction

Lentiviral transduction of Ngn2-neural progenitors was performed at Day –1 with medium change to DMEM/F12 containing 4 μl/ml viral solution. At Day 0, 24 h after transduction, cells were washed once for 30 min with pre-warmed Neurobasal Medium containing all factors to remove lentiviral particles and thus prevent transduction of astrocytes during subsequent plating. In a second-generation lentiviral backbone, the expression of 2N4R FTDtau with an in-frame EGFP or mCherry C-terminal fluorescent tag was driven by a cytomegalovirus promoter. The following FTDtau mutants were used: P301L (FTDtau^1^), S320F (FTDtau^2^), and P301L + S320F (FTDtau^1+2^). Lentiviral particles were generated as previously described (Naldini et al., 1996).

### Tau seeding

PFFs of K18 P301L tau came from a study that was previously published (Wiersma et al., 2019). Briefly, PFFs were generated *in vitro* by incubation of 40 μM recombinant K18 P301L tau in 100 mM sodium acetate (Sigma) buffer (pH 7.0) for 24 h at 37°C in the presence of 10 μM low-molecular-weight heparin (Thermo Fisher) and 2 mM DTT (Sigma). PFFs were diluted to 10 μM in sodium acetate buffer, sonicated using a probe sonicator (25 pulses at 50% power for 24–30 microns peak to peak, with 3 sec rest every 5th pulse), and stored at −80°C until use. PFFs or an equal volume of sodium acetate buffer as vehicle control were mixed with complete medium and added at a final concentration of 75 nM to co-cultures with medium refreshment at Week 2.

### Tau AON design and delivery

AON design was based on a previous study (DeVos et al., 2017) and AONs were synthesized and purified by Eurogentec. Briefly, 20 nucleotide gapmer AONs contained a phosphorothioate backbone with a central unmodified region of 10 oligodeoxynucleotides flanked by 5 nucleotides on the 5′ and 3′ termini containing 2′-O-methoxyethyl modifications. AONs used in Supplementary Figure S10A–C contained additional 5′ Cy3 and 3′ cholesteryl-triethylene glycol modifications. After synthesis, AONs were purified using reverse-phase high-performance liquid chromatography and desalted on a Sephadex G25 column. AONs were reconstituted to 100 μM in sterile Milli-Q water and stored at −20°C until use. For lipofection, AONs were incubated for 10 min at room temperature (RT) with pre-incubated Lipofectamine 3000 (Invitrogen) at a concentration of 0.5 μl/μg AON in Neurobasal Medium without factors, subsequently mixed 1:10 with complete medium, and added to the co-cultures with medium refreshment. Final AON concentration at 100 nM was used unless otherwise specified. MAPT AON sequence: 5′-GCTTTTACTGACCATGCGAG-3′; scramble AON sequence: 5′-CCTTCCCTGAAGGTTCCTCC-3′.

### ISR and ROS assay

To activate the ISR via the induction of ER-stress, co-cultures were treated with TM (10 μg/ml; Sigma) or an equal volume of DMSO (Sigma) as vehicle control for 48 h prior to fixation. To generate ROS, co-cultures were treated with Men (100 μM; Sigma) or an equal volume of DMSO as vehicle control for 4 h at 37°C/5% CO_2_. To determine ROS levels, cells were loaded with CellROX green reagent (5 μM; Thermo Fisher) during the last 30 min of incubation. To prevent photobleaching of CellROX green, co-cultures were processed for immunofluorescent staining and imaged by automated microscopy within 24 h.

### Immunocytochemistry

The fixation protocols were adapted from Wiersma et al. (2019) with slight modifications. Briefly, paraformaldehyde (PFA) fixation consisted of 20 min incubation at RT in 2% PFA (Electron Microscopy Sciences) by addition of 4% PFA in PBS (pH 7.4) to the same volume of culture medium, followed by fixation in 4% PFA for 20 min at RT. For MeOH fixation, cells were fixed for 20 min in ice-cold 100% MeOH. PFA- and MeOH-fixed cultures were washed once with PBS for 15 min and immediately processed for immunostaining or stored at 4°C. For immunostaining, cells were blocked and permeabilized for 3 h in blocking solution, i.e. 5% normal goat serum (Thermo Fisher), 0.1% bovine serum albumin (Sigma), and 0.3% Triton X-100 (Sigma) in PBS (pH 7.4), and subsequently incubated with primary antibodies diluted in blocking solution for 1 h at RT followed by overnight at 4°C. After washing three times in PBS, cells were incubated for 3 h at RT with Alexa Fluor-conjugated secondary antibodies (488, 546, 568, and 647; Invitrogen) diluted 1:1000 in blocking solution. Alexa Fluor 647-conjugated phalloidin (1:1000; Invitrogen, A22287) and HS169 (1:500; a kind gift of Peter Nilsson) were used during secondary antibody incubation to label actin and stacked beta-sheets (Shirani et al., 2015), respectively. Cells were washed three times in PBS for 15 min at RT, and cell nuclei were stained with 4′,6-diamidino-2-phenylindole (DAPI; 2.5 μg/ml; Sigma) in PBS of the second wash. Confocal images were acquired with a Nikon Eclipse Ti confocal microscope equipped with a 10× air and 40× oil immersion objective and controlled by NisElements 4.30 software (Nikon). Either single focal planes or Z-stacks with a step size of 1 or 4 μm were obtained and each parameter, including laser power and detector gain, was kept constant within each experiment for valid comparison. The following primary antibodies were used: ATF4 (1:250; Cell Signaling Technology, D4B8 118155, RRID:AB_2616025), CD44 (1:100; DSHB, H4C4, RRID:AB_528147), MAP2 (1:5000; Millipore, AB5543, RRID:AB_303248), MC1 (1:500; a kind gift of Peter Davies), PSD95 (1:200; Abcam, AB2723, RRID:AB_303248), pTau-AT8 (Ser202, Thr205; 1:200; Thermo Fisher, MN1020, RRID:AB_223647), pTau-AT100 (Thr212, Ser214; 1:200; Thermo Fisher, MN1060, RRID:AB_223652), Synaptophysin 1 (1:1000; Synaptic Systems, 101004, RRID:AB_1210382), and Tuj1 (1:1000; R&D Systems, MAB1195, RRID:AB_357520). The pTau-AT8, pTau-AT100, and MC1 antibody concentrations were adapted from Wiersma et al. (2019). Maximum intensity-projected Z-stacks are shown. ImageJ software (National Institutes of Health) was used to adapt images for publication.

### High-content microscopy analysis

High-content microscopy was performed on a CellIInsight CX7 HCS platform (Thermo Fisher) in widefield mode with a 20× objective. DAPI fluorescence was used for autofocus, and Z-stacks of 20 μm with a step size of 4 μm were obtained. Either 16 or 36 fields per well were acquired. Maximum intensity projections were analysed with Columbus analysis software (v2.5.2.124862; PerkinElmer) by in-house developed scripts adapted from Rosato et al. (2021) with modifications. Briefly, based on DAPI, MAP2, and SYP1 staining, quantifications related to neuron and astrocyte number, neuronal morphology, and presynapses were extracted (see Supplementary Figure S1). Nuclei touching the border of the image field were excluded for analysis. Neuronal nuclei are distinguished from astrocytic nuclei by their relatively smaller size, higher DAPI intensity, and larger MAP2 overlap in the soma. It is important to note that *in vitro*, neuronal nuclei are smaller than astrocytic nuclei. Astrocytic nuclei are obtained by subtracting the number of neuronal nuclei from the total number of nuclei. Based on MAP2 staining, dendrites were traced using the built-in CSIRO neurite analysis module. Morphological measures (dendrite length, primary dendrites, extremities, segments, and branch points) per neuron are obtained by normalization against the number of neuronal nuclei. SYP1-positive presynapses were detected using local intensity maxima within the MAP2-positive dendrite trace, and the total number of puncta was normalized for dendrite length to yield presynapse density. A threshold (≥0.4) was applied to the MAP2 channel to quantify mean fluorescence intensity and SD to assess tau levels and aggregation, respectively. The fluorescence intensity of tau in co-cultures was background-corrected by subtracting the mean fluorescence intensity of untransduced co-cultures. The protocol to quantify nuclear CellROX green and ATF4 was adapted from van Ziel et al. (2020) and Wolzak et al. (2022) with slight modifications. Briefly, nuclear intensity of CellROX green and ATF4 in neurons and astrocytes was background-corrected by subtracting the mean fluorescence intensity in a 2.5 μm-restricted ring region around the nucleus from the mean fluorescence intensity within the nucleus. Wells with excessive cell clustering were not included in the analysis.

### Immunoblot analysis of sarkosyl extracts

Co-cultures (Day –2: 1.2 × 10^5^ Ngn2-hiPSCs; Day 0: 1.2 × 10^5^ astrocytes) in 6-well plates were washed in ice-cold PBS and scraped into ice-cold high-salt buffer (10 mM Tris-HCl, pH 7.4, 0.8 M NaCl, 1 mM EDTA, 2 mM DTT, 0.5 mM PMSF, and protease and phosphatase inhibitor cocktail (both Roche)) with 0.1% sarkosyl (Sigma) at Week 4. Lysates were centrifuged for 5 min at 2000 rcf to remove cellular debris and separated into sarkosyl-soluble and insoluble fractions by three sequential ultracentrifugation steps at 1.0 × 10^5^ rcf for 30 min at RT as previously described (Wiersma et al., 2019). Briefly, after the first ultracentrifugation, the supernatant was kept as sarkosyl-soluble fraction, and the pellet was washed in sarkosyl-containing high-salt buffer and ultracentrifuged again. The resulting pellet was washed and resuspended in 1% sodium dodecyl sulfate (SDS) in PBS and ultracentrifuged, and the supernatant was kept as sarkosyl-insoluble fraction. Protein concentration of the sarkosyl-soluble fractions was determined using BCA assay. All samples were diluted in SDS loading buffer and boiled for 5 min at 96°C. Then, 10 μg of proteins from the sarkosyl-soluble and threefold more volume of the corresponding sarkosyl-insoluble fraction were loaded per lane of a 4%–15% gradient Mini-Protean TGX stain-free precast polyacrylamide gel and transferred onto nitrocellulose membranes (both Bio-Rad). Membranes were cut ∼45 kDa and blocked with 5% (*w*/*v*) skimmed milk powder (Millipore) in Tris-buffered saline containing 0.05% Tween (TBS-T) (Millipore). Upper parts of the membranes were incubated with Tau-5 (1:1000; Abcam, AB80579, RRID:AB_1603723) and lower parts with GAPDH (1:2500; Merck, MAB374, RRID:AB_2107445) primary antibodies diluted in blocking buffer overnight at 4°C. After three TBS-T washes, membranes were incubated with HRP-conjugated secondary antibodies (DAKO) diluted 1:2000 in blocking buffer for 1 h at RT, washed in TBS-T, and developed using SuperSignal West Femto substrate (Thermo Fisher). Membranes were scanned using the Odyssey Fc system and visualized using Image Studio 5.2 software (both LI-COR).

### Extracellular tau assay

Conditioned media were removed from neuron–astrocyte co-culture after 8 weeks and centrifuged at 2000 rcf for 3 min to remove cellular debris and frozen at –20°C until use. Tau levels were quantified using a singleplex total tau assay (MSD S-Plex, K151AGPS-1) as per manufacturer's instructions. Briefly, plate wells were coated and blocked, and samples were diluted 1:32 except for two experimental replicates in the FTDtau^1^ group that exceeded upper detection limit and were therefore diluted 1:200. Per sample, duplicates were loaded and concentration was acquired by back-fitting to the calibration curve. Samples were quantified in two runs, and same samples with different dilutions as well as cerebrospinal fluid samples with known tau concentrations were included to ensure <15% inter-assay variation.

### Statistics

High-throughput quantification of co-cultures was performed in a blinded manner by automated analysis. Graphpad Prism version 8.4.2 (Graphpad software RRID:SCR_002798) was used to perform statistical analysis and graphing. Shapiro–Wilk test was used for normality testing and outliers were excluded from analysis using the ROUT method (*Q* = 1%) in Graphpad Prism. Nested T test was used for statistical analyses of two groups. Nested one-way analysis of variance (ANOVA) or two-way ANOVA with Tukey's post-hoc test was used for multiple comparisons. Unless otherwise specified, graphs show mean ± standard error of the mean (SEM) and data points represent the mean/well. Where applicable, the type of data normalization is indicated in the figure legend. Correlation in Supplementary Figure S4D was analysed by Pearson correlation analysis. Linear regression analysis was used for comparisons of morphological development in Supplementary Figure S6B–G. A *P-*value <0.05 was considered statistically significant. For each dataset, the statistical test and exact significance values are given in Supplementary Table S1 and significance is shown in the graphs as *P < 0.05, **P < 0.01, ***P < 0.001, and ****P < 0.0001. ns, not significant.

### Availability of data and materials

The data that support the findings of this study are available from the corresponding author upon reasonable request.

## Supplementary Material

mjac071_Supplemental_File
