# Supplementary material for: Intraneuronal tau aggregation induces the integrated stress response in astrocytes
Source: J Mol Cell Biol. 2022 Dec 15;14(10):mjac071. doi: 10.1093/jmcb/mjac071 (PMC10080549; doi:10.1093/jmcb/mjac071)
Supplement: mjac071_Supplemental_File [file mjac071_supplemental_file.pdf]

Intraneuronal tau aggregation induces the integrated stress response in astrocytes

Kevin L. Batenburg, Nael N. Kasri, Vivi M. Heine & Wiep Scheper*

*Correspondence to: Wiep Scheper

w.scheper@amsterdamumc.nl

Supplementary Material

Supplementary Figure S1 – S11

Supplementary Table S1

Supplementary Figure S1

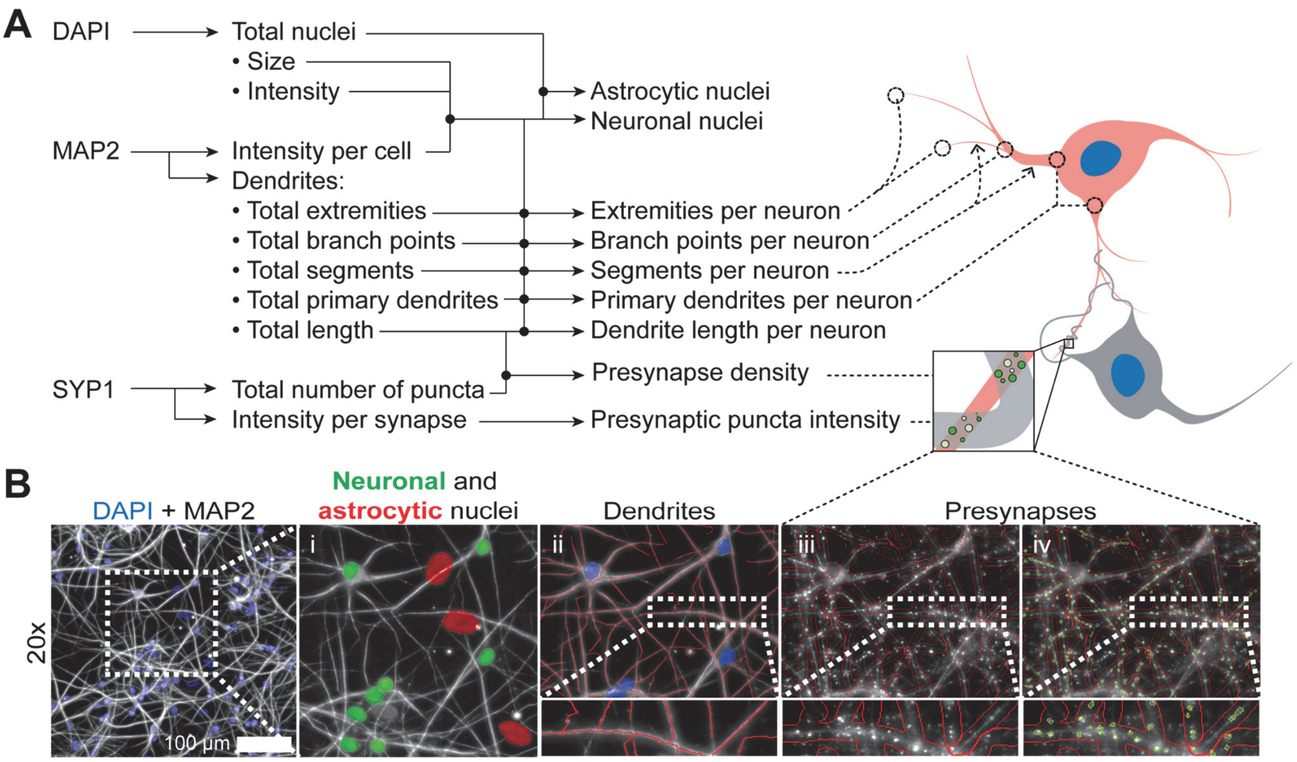

High-content microscopy analysis workflow for nuclear, morphological and presynaptic measurements.

(**A**) Schematic representation of the high-content screening and analysis workflow to distinguish neurons and astrocytes in human co-culture and to quantify parameters of neuronal morphology and presynapses, based on DAPI, MAP2 and SYP1 (immuno)staining. Neuronal nuclei are distinguished from astrocytic nuclei by their relatively smaller size, higher DAPI intensity and larger MAP2 overlap in the soma, as is also shown in Figure 1C. Astrocytic nuclei are obtained by subtracting the number of neuronal nuclei from the total number of nuclei. By normalization against neuronal nuclei, morphology measures per neuron are obtained, and the number of presynaptic puncta are normalized for dendrite length to calculate presynapse density. See *Materials and Methods* for more detail. (**B**) Representative widefield image of co-cultures at week 4 imaged at 20x magnification. Neurons and astrocytes are distinguished (MAP2-positive (neuronal) nuclei in green; MAP2-negative (astrocytic) nuclei in red) (**i**) and dendrites are detected (traces in red) (**ii**). Presynapses that fall within a dendrite region (red outline) (**iii**) are detected (selected puncta in green) (**iv**).

Supplementary Figure S2

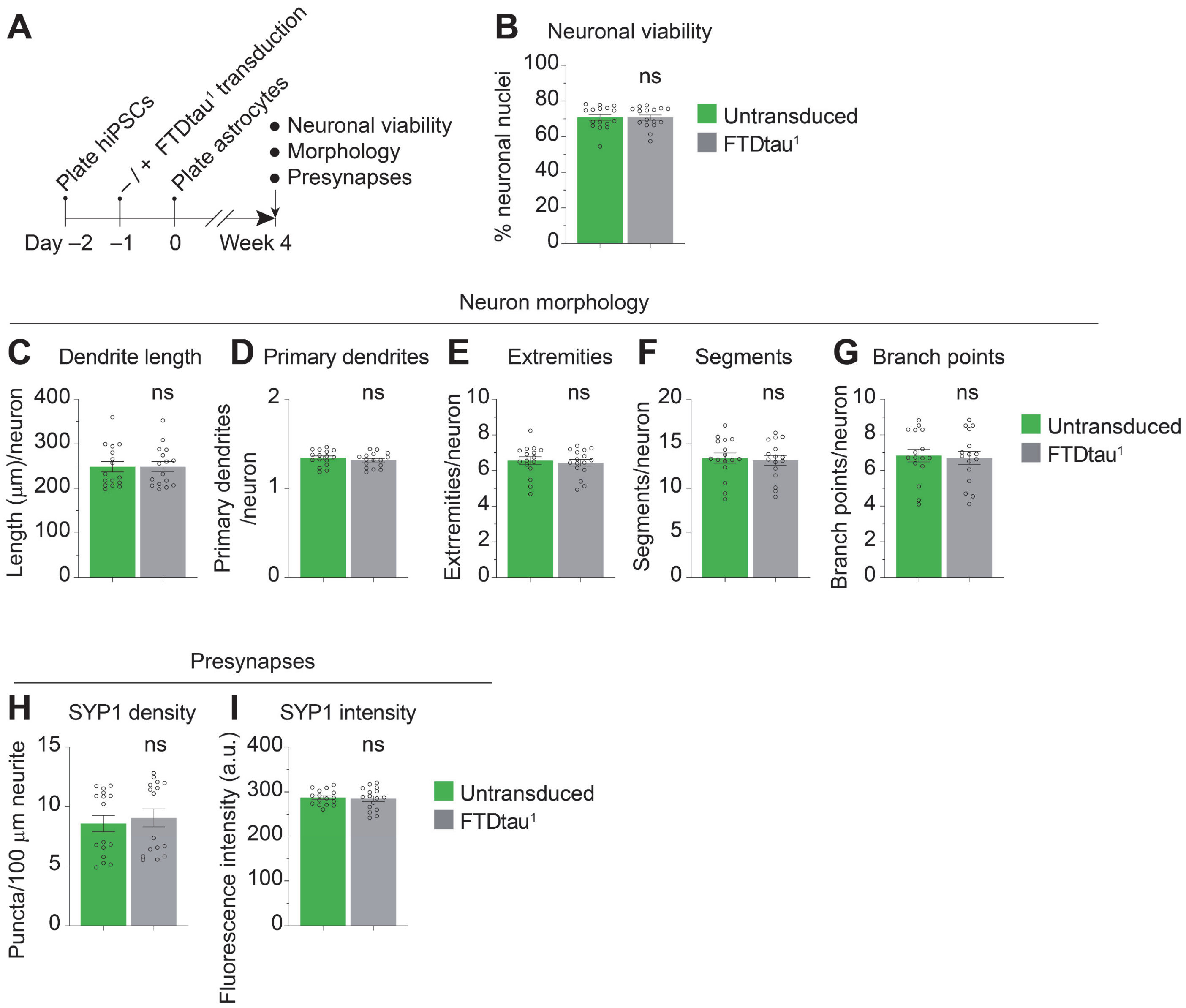

Tau overexpression does not affect neuronal viability, morphology and presynapses.

Neuronal viability, morphology and presynapses were quantified by automated microscopy (see Supplementary Figure S1) in FTDtau[1]-transduced and untransduced co-cultures at 4 weeks. (**A**) Schematic representation of the experiment. (**B**) Neuronal viability as determined by the percentage of neurons in co-cultures at 4 weeks, without (green) or with transduction with EGFP-tagged FTDtau[1] (grey). (**C**-**G**) Parameters of neuron (MAP2) morphology in co-cultures at 4 weeks, without (green) or with transduction with EGFP-tagged FTDtau[1] (grey) as determined by dendrite length (**C**), primary dendrites (**D**), extremities (**E**), segments (**F**), and branch points (**G**). (**H** and **I**) Quantification of presynapses (SYP1) in co-cultures at 4 weeks, without (green) or with transduction of EGFP-tagged FTDtau[1] (grey) as determined by density (**H**) and fluorescence intensity (**I**). Bar graphs show the mean ± SEM, data points represent mean/well. *N* = 4 independent experiments, *n* = 14 wells were analysed for **B**-**I**. Statistical significance was assessed by Nested T test. *ns* not significant. For each dataset, Supplementary Table S1 lists the exact p values and total number of cells analysed.

Supplementary Figure S3

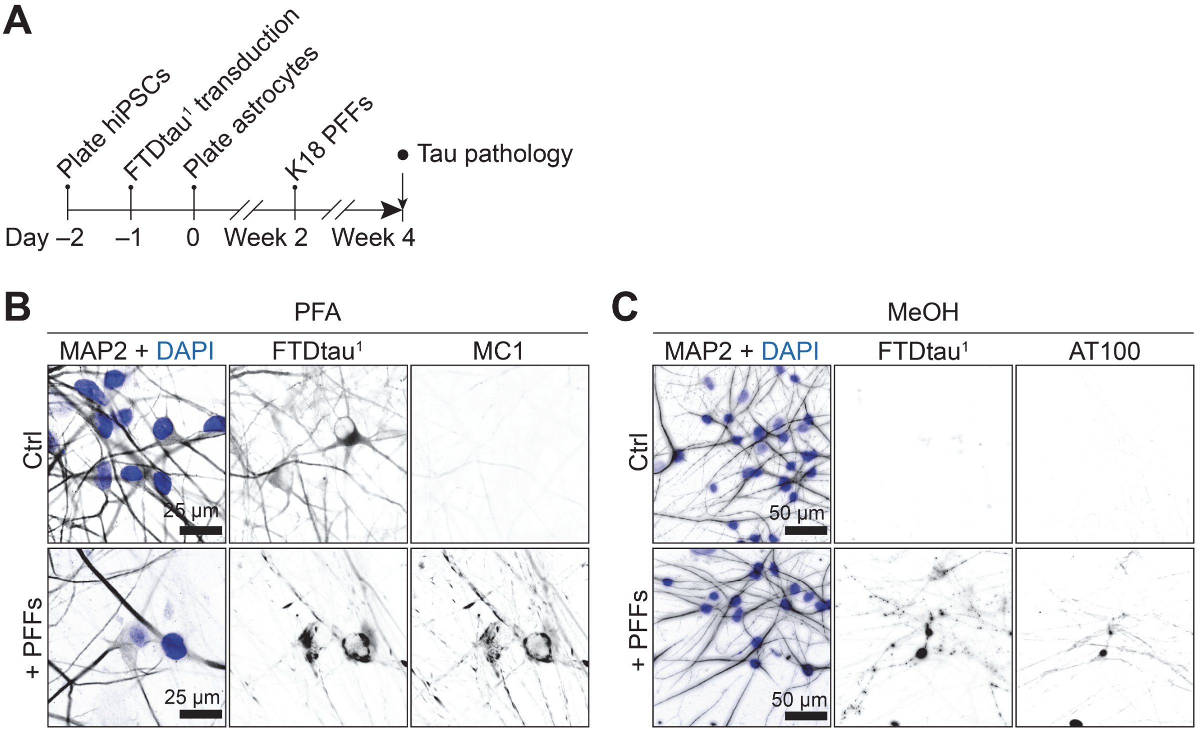

FTDtau[1] does not aggregate in the absence of exogenous seeds.

Seeding of FTDtau[1]-transduced co-cultures was performed at 2 weeks by addition of tau PFFs to the culture medium, and tau aggregation was assessed at week 4. (**A**) Schematic outline of the experiment. (**B** and **C**) Representative confocal (**B**) and widefield (**C**) images of co-cultures expressing EGFP-tagged FTDtau[1] treated with 75 nM K18 tau PFFs or vehicle control (Ctrl). (**B**) EGFP direct fluorescence and immunostainings for MAP2 and a pathological conformation of tau (MC1) are shown in greyscale. Cell nuclei were visualized using DAPI (blue). (**C**) Soluble FTDtau[1] was removed by MeOH fixation. EGFP direct fluorescence and immunostainings for MAP2 and pathologically phosphorylated tau (AT100) are shown in greyscale. Cell nuclei were visualized using DAPI (blue).

Supplementary Figure S4

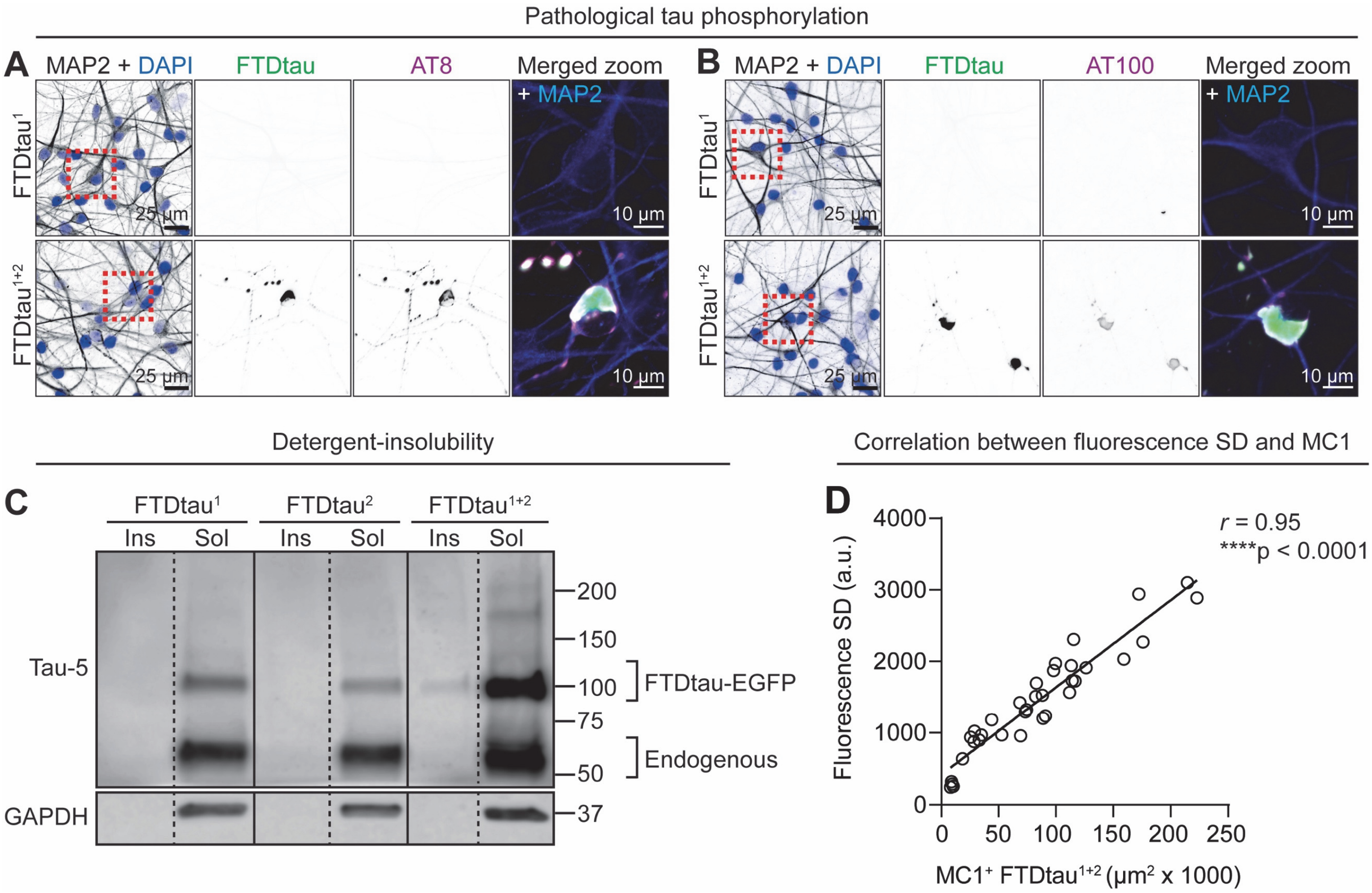

Double mutant FTDtau^{1+2} but not single mutants FTDtau^{1} and FTDtau^{2} spontaneously forms aggregates

Co-cultures were transduced with EGFP- tagged FTDtau^{1} or FTDtau^{1+2} and tau aggregation was assessed at week 4. (**A** and **B**) Representative confocal images of neuron-astrocyte co-cultures, transduced with EGFP-tagged FTDtau^{1} or FTDtau^{1+2}. At 4 weeks, soluble FTDtau was removed by MeOH fixation and co-cultures were subsequently stained for MAP2 and pathologically phosphorylated tau with AT8 (**A**) or AT100 (**B**), single channels are shown in greyscale. Cell nuclei were visualized by DAPI (blue). Zooms of the boxed areas in the MAP2+DAPI panels are shown as merged image with MAP2 (blue), FTDtau^{1} or FTDtau^{1+2} (green) and AT8 or AT100 (magenta). (**C**) Western blot of the sarkosyl-soluble (Sol) and insoluble (Ins) fractions of lysates from EGFP-tagged FTDtau^{1}, FTDtau^{2} and FTDtau^{1+2}-expressing co-cultures. Membranes were immunoblotted for total tau (Tau-5) and GAPDH as loading and fractionation control. The position of overexpressed FTDtau-EGFP and endogenous tau is indicated. (**D**) Correlation analysis between the SD of EGFP fluorescence and the density of pathological (MC1-positive) FTDtau^{1+2} in co-cultures expressing EGFP-tagged FTDtau^{1+2} at 4 weeks. $N = 4$ independent experiments, $n = 34$ wells. A linear regression line is shown and data points represent the mean value of individual wells. After Shapiro-Wilk normality testing, correlations were analysed by Pearson correlation analysis. Correlation coefficient (r) and exact p value are shown in the graph. Supplementary Table S1 lists the total number of cells analysed.

Supplementary Figure S5

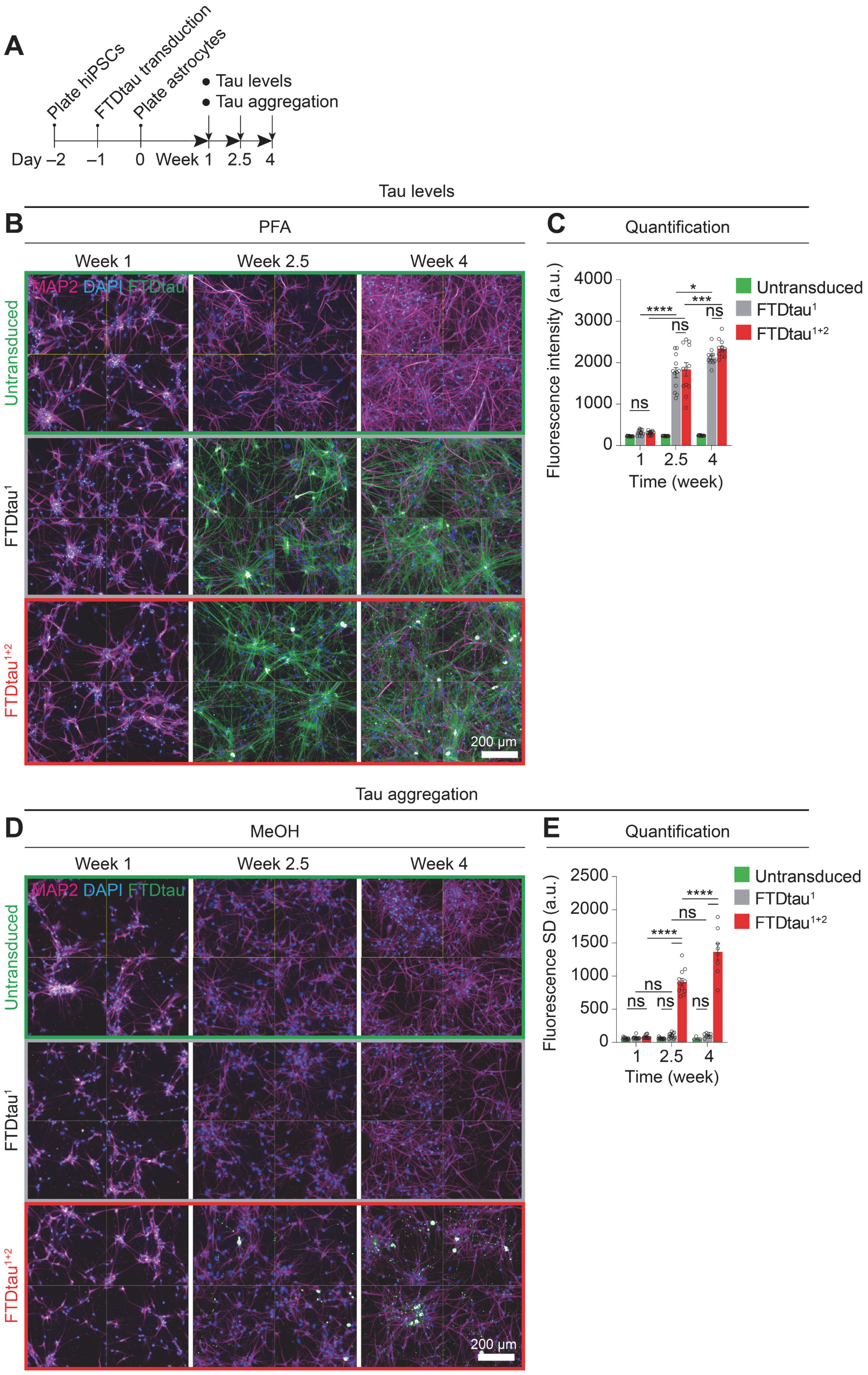

◄ **Timing and specificity of FTDtau^{1+2} aggregation**

Levels of total and aggregated tau were quantified in untransduced and EGFP-tagged FTDtau^{1}- or FTDtau^{1+2}-transduced co-cultures at week 1, 2.5 and 4. (**A**) Schematic representation of the protocol to measure intraneuronal tau levels and aggregation in a human neuron-astrocyte co-culture over time. Representative widefield images captured by automated microscopy at week 1, 2.5 and 4 of PFA-fixed (**B**) and MeOH-fixed (**D**) co-cultures without or with transduction for EGFP-tagged FTDtau^{1}- or FTDtau^{1+2}. Each panel consists of 4 widefield images, showing EGFP direct fluorescence (green), immunostainings for MAP2 (magenta) and cell nuclei visualized using DAPI (blue). (**C** and **E**) Quantification of EGFP mean fluorescence intensity from **B** showing total tau levels and EGFP mean fluorescence standard deviation (SD) from **D** showing aggregated tau levels, respectively, in untransduced (green), FTDtau^{1}- (grey) or FTDtau^{1+2}- (red) transduced co-cultures at week 1, 2.5 and 4. Bar graphs show the mean ± SEM, data points represent mean/well. N = 3 independent experiments, n = 12 wells and $N \geq 2$ independent experiments, n = 8 wells were analysed for **C** and **E**, respectively. Shapiro-Wilk normality testing followed by two-way ANOVA with Tukey's post-hoc analysis. *p<0.05, ***p<0.001, ****p<0.0001, *ns* not significant. Supplementary Table S1 lists the exact p values and total number of cells analysed.

Supplementary Figure S6

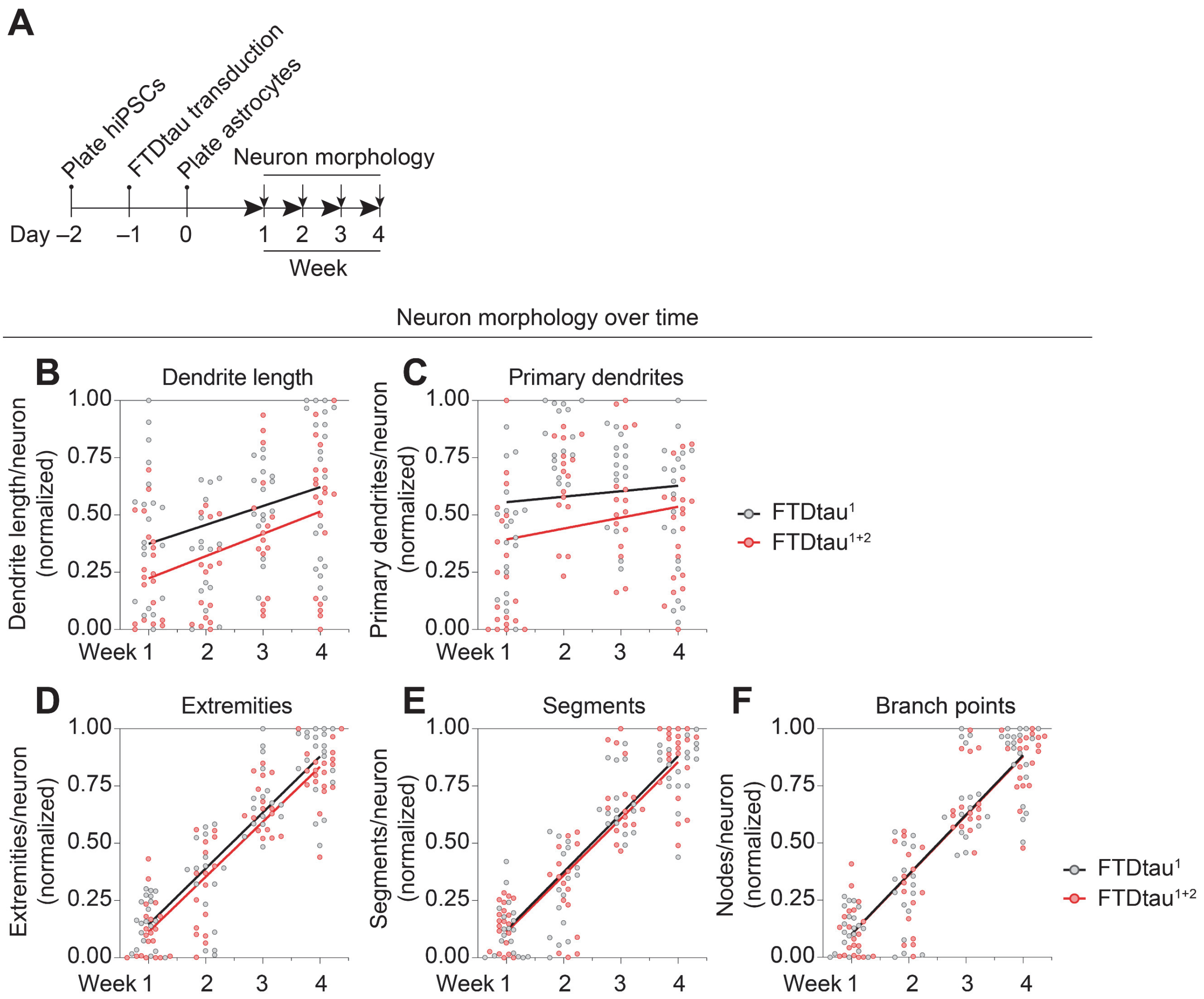

G

	vs. week:	Dendrite length	Primary dendrites	Extremities	Segments	Branch points
FTDtau[1]	R^2	0.1131	0.01044	0.7778	0.7838	0.7877
	P value	0.0039	ns	<0.0001	<0.0001	<0.0001
FTDtau[1+2]	R^2	0.1729	0.03914	0.7973	0.7933	0.8038
	P value	0.0003	ns	<0.0001	<0.0001	<0.0001
	Slopes different?	No	No	No	No	No

Tau aggregation does not affect the morphological development of neurons.

Co-cultures were transduced for EGFP-tagged FTDtau[1] or FTDtau[1+2] and immunostained for MAP2 to assess neuron morphology at week 1, 2, 3 and 4 by high-content microscopy. (**A**) Schematic representation of the experiment. (**B**-**F**) Morphological analysis of neurons transduced with EGFP-tagged FTDtau[1] (grey) or FTDtau[1+2] (red) at week 1, 2, 3 and 4 by quantification of dendrite length (**B**), primary dendrites (**C**), extremities (**D**), segments (**E**) and branch points (**F**) per neuron. Linear regression lines are shown in each graph and data points represent the mean/well. The lowest and highest value per experiment were set to 0 and 1, respectively. Week 1 and 4: N = 5 independent experiments, n = 20 wells. Week 2 and 3: N = 4 independent experiments, n = 16 wells. Two-way ANOVA with Tukey's post-hoc test confirmed statistical differences within but not between groups. Supplementary Table S1 lists the exact p values and total number of cells analysed. (**G**) Table showing the R^2 and corresponding p value for the linear regression of each morphological parameter versus weeks in co-culture. None of the regression slopes of FTDtau[1] and FTDtau[1+2] are significantly different.

Supplementary Figure S7

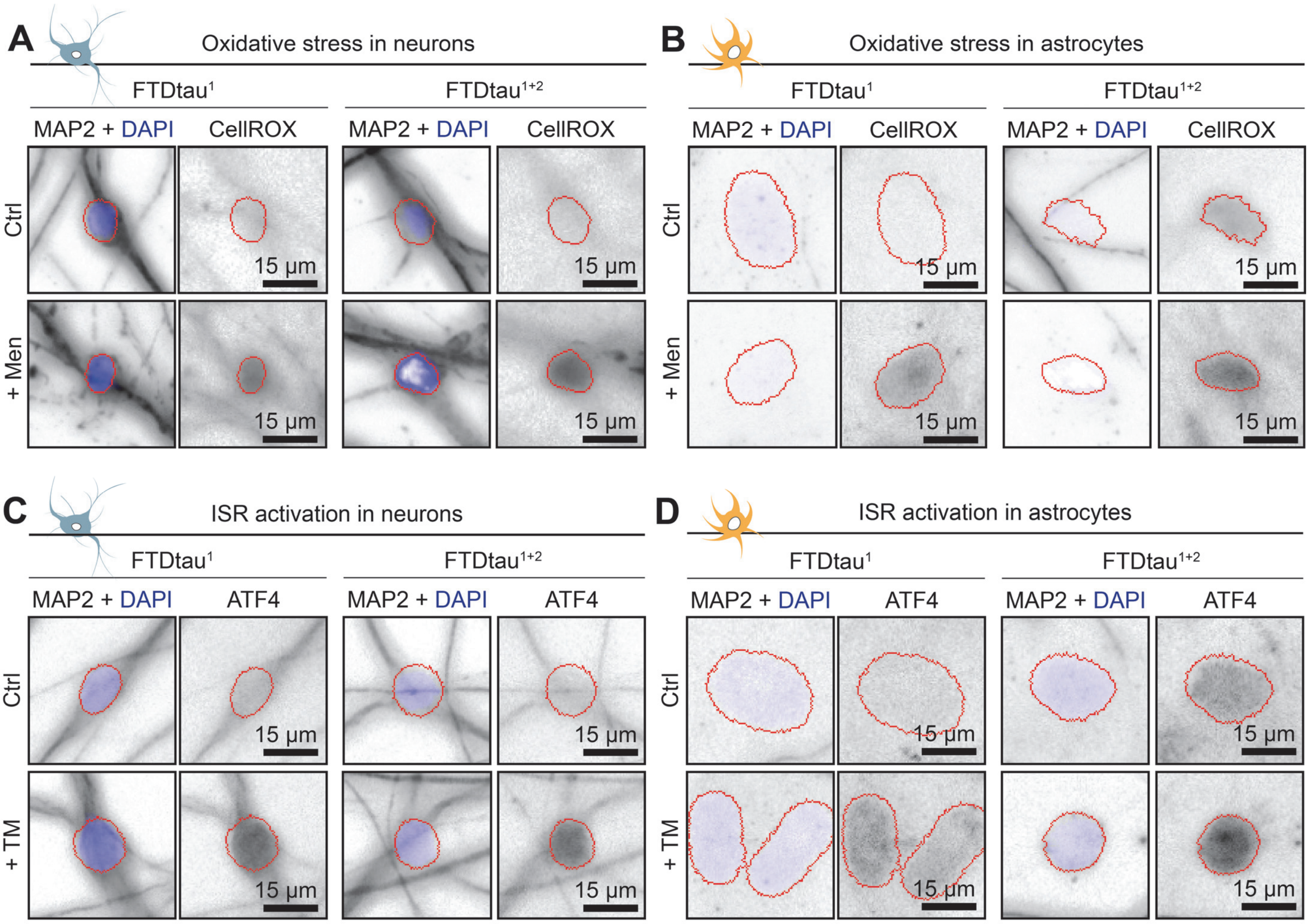

Neuronal FTDtau^{1+2} induces increased nuclear CellROX green fluorescence and ATF4 immunoreactivity in astrocytes at 8 weeks of co-culture.

Co-cultures were transduced with mCherry- (**A** and **B**) or EGFP- tagged (**C** and **D**) FTDtau^{1} or FTDtau^{1+2} and oxidative stress and ISR activation were measured in neurons and astrocytes at 8 weeks. Shown are representative zoomed widefield images captured by automated microscopy of co-cultures showing MAP2 immunostaining (grey) and nuclei visualized by DAPI (blue) that were detected by automated microscopy (red outline). The ROS probe CellROX green (**A** and **B**) and ISR target ATF4 (**C** and **D**) were detected by direct- and immunofluorescence, respectively, in the nuclei of neurons (**A** and **C**) and astrocytes (**B** and **D**), in the absence (Ctrl) or presence of menadione (Men) or tunicamycin (TM).

Supplementary Figure S8

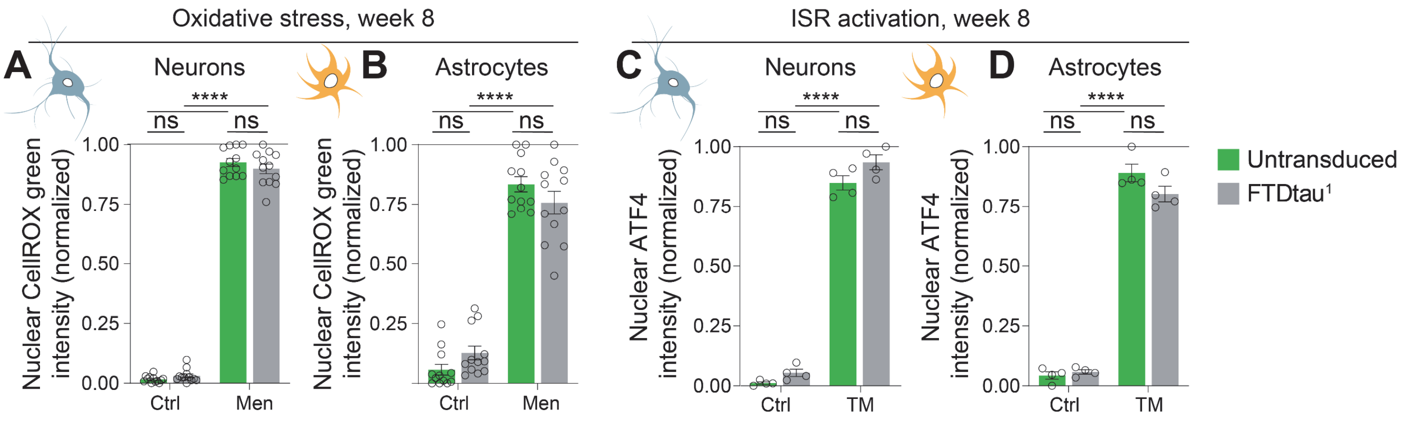

Tau overexpression does not induce oxidative stress and ISR activation in neurons and astrocytes

Oxidative stress (**A** and **B**) and ISR activation (**C** and **D**) were quantified in neurons and astrocytes by automated microscopy in FTDtau[1]-transduced and untransduced co-cultures at 8 weeks. (**A** and **B**) Oxidative stress in co-cultures without (green) or with transduction with mCherry-tagged FTDtau[1] (grey), treated with 100 µM menadione (Men) or vehicle control (Ctrl) 3.5 hours before loading with CellROX green. Immunostaining was performed for MAP2, cell nuclei were visualized using DAPI. Oxidative stress was determined by quantifying CellROX green intensity in neuronal (**A**) and astrocytic (**B**) nuclei. (**C** and **D**) ISR activation in co-cultures without (green) or with transduction with EGFP-tagged FTDtau[1] (grey), treated 48 hours with 10 µg/mL tunicamycin (TM) or vehicle control (Ctrl). Immunostaining was performed for MAP2 and ATF4, cell nuclei were visualized using DAPI. ISR activation was determined by quantifying ATF4 intensity in neuronal (**C**) and astrocytic (**D**) nuclei. Bar graphs show the mean ± SEM, data points represent mean/well. The lowest and highest value per experiment were set to 0 and 1, respectively. $N = 3$ and $N = 1$ independent experiments, $n = 12$ and $n = 4$ wells were analysed for **A, B** and **C, D**, respectively. After Shapiro-Wilk normality testing, statistical analysis was by two-way ANOVA and Tukey's post-hoc analysis. ****p<0.0001, *ns* not significant. Supplementary Table S1 lists the exact p values and total number of cells analysed.

Supplementary Figure S9

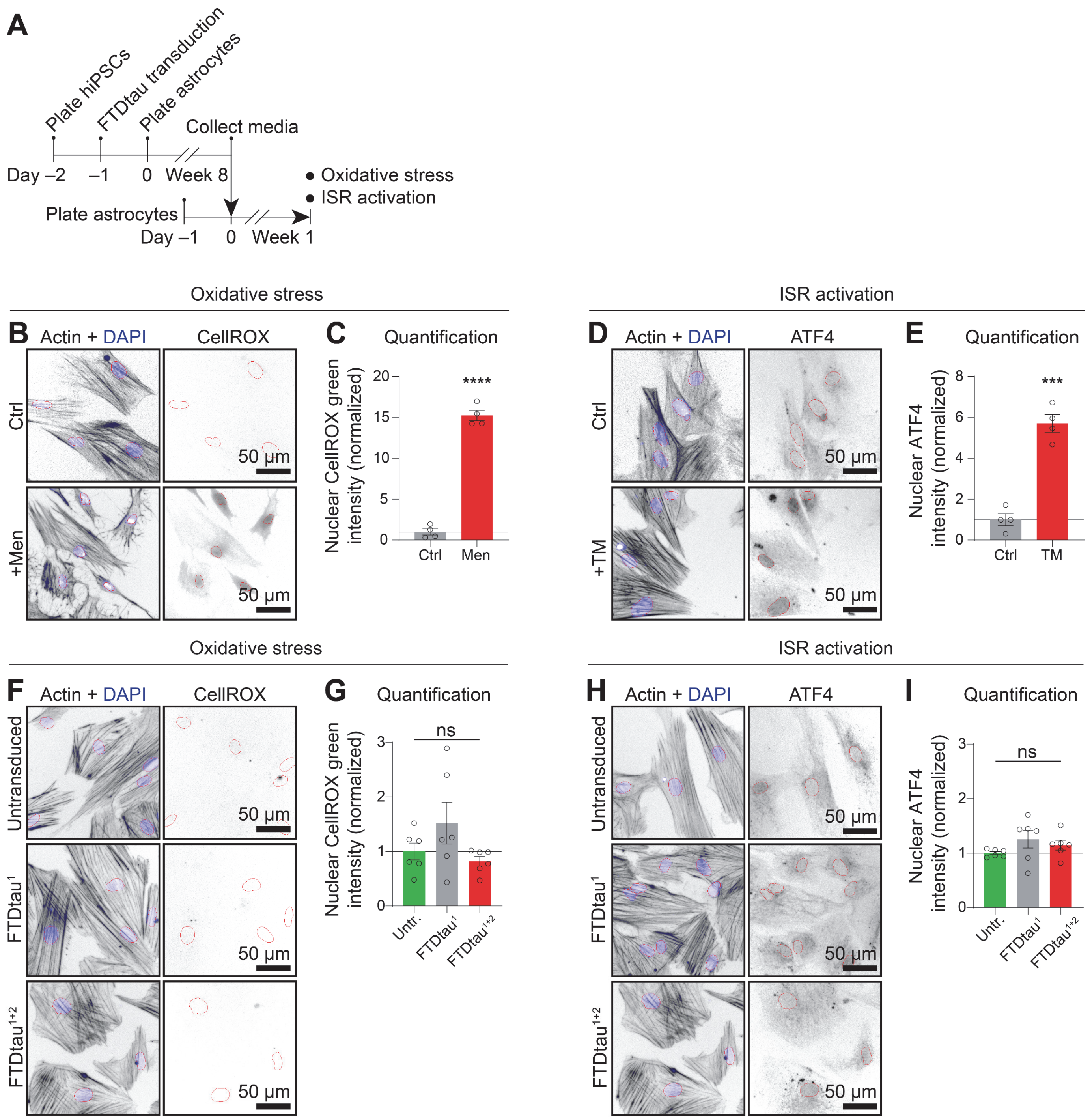

Oxidative stress and ISR activation in astrocytes by intraneuronal tau aggregation is not transferred via conditioned co-culture media.

Monocultured astrocytes were exposed to conditioned media of 8 week-old untransduced co-cultures, or co-cultures expressing EGFP- tagged FTDtau[1] or FTDtau[1+2]. After one week, oxidative stress and ISR activation were measured by automated microscopy using CellROX green fluorescence and ATF4 immunofluorescence, respectively. (**A**) Schematic representation of the experiment. (**B-E**) Monocultured astrocytes in unconditioned media in the absence (Ctrl, grey) or presence of menadione (Men, red) (**B**) or tunicamycin (TM, red). (**B** and **D**) Representative zoomed widefield images captured by automated microscopy showing actin (phalloidin; grey) and nuclei visualized by DAPI (blue) that were detected by the analysis (red outline) as a merged image, and CellROX green (**B**) and ATF4 (**D**) are shown as separate

◄ (*Legend Sup. Fig. S9 continued*) greyscale images. Automated quantification of CellROX green and ATF4 nuclear fluorescence in **B** and **D** is shown in (**C**) and (**E**)**,** respectively. (**F**-**I**) Monocultured astrocytes exposed to conditioned media of untransduced (green) co-cultures or co-cultures expressing EGFP- tagged $FTDtau^{1}$ (grey) or $FTDtau^{1+2}$ (red). Representative zoomed widefield images captured by automated microscopy showing actin (phalloidin; grey) and nuclei visualized by DAPI (blue) that were detected by the analysis (red outline) as a merged image, and CellROX green (**F**) and ISR target ATF4 (**H**) are shown as separate greyscale images. Automated quantification of CellROX green and ATF4 nuclear fluorescence in **F** and **H** is shown in (**G**) and (**I**)**,** respectively. For **C, E**, **G** and **I** mean values of vehicle and untransduced controls were set to 1, respectively. N = 1**,** n = 4 and N = 3, n = 6 independent experiments and wells were analysed for **C, E** and **G, I**, respectively. Bar graphs show the mean ± SEM, data points represent mean/well. After Shapiro-Wilk normality testing, significance was assessed using Student's T-test (**C** and **E**) and Nested One-way ANOVA with Tukey's post-hoc analysis (**G** and **I**). ***p=0.001, ****p<0.0001, *ns* not significant. Supplementary Table S1 lists the exact p values and total number of cells analysed.

Supplementary Figure S10

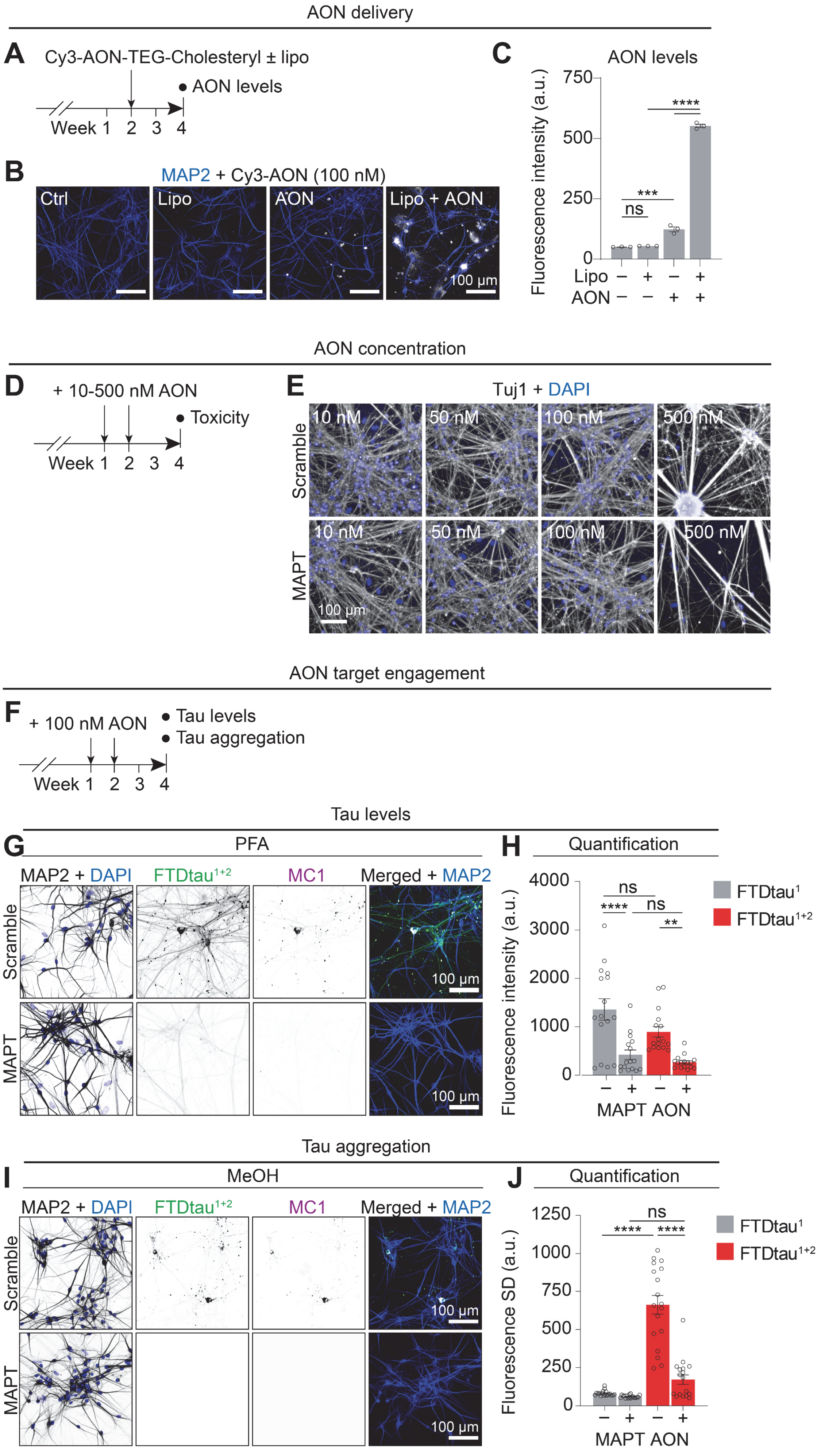

◄ **MAPT antisense therapy prevents tau aggregation in human co-culture.**

Untransduced co-cultures were treated at week 2 with 100 nM MAPT AON conjugated to a 3' cholesteryl group with a triethylene glycol (TEG) linker and to a 5' Cy3 fluorescent marker, in the absence or presence of 0.5 µL lipofectamine (Lipo) per µg MAPT AON, and fixed at week 4. (**A**) Schematic representation of the experiment. (**B**) Representative confocal images of 4 week-old co-cultures treated with MAPT AON in the absence or presence of 0.5 µL Lipo per µg MAPT AON, showing direct Cy3 fluorescence (grey) and immunostaining of MAP2 (blue). (**C**) Quantification of Cy3 mean fluorescence within a MAP2-positive mask at week 4 in untransduced co-cultures that were either untreated or treated with MAPT AON in the absence or presence of Lipo. *N* = 1 independent experiment, *n* = 3 wells. Bar graphs show the mean ± SEM, data points represent mean/well. Shapiro-Wilk normality testing followed by two-way ANOVA with Tukey's post-hoc analysis. ***p<0.001, ****p<0.0001, *ns* not significant. (**D** and **E**) To titrate lipofection of MAPT AON, untransduced co-cultures were treated at week 1 and 2 with 10, 50, 100 or 500 nM unconjugated scramble or MAPT AON in the presence of 0.5 µL lipofectamine per µg AON and fixed at week 4. (**D**) Schematic representation of the experiment. (**E**) Representative confocal images of co-cultures treated with 10-500 nM scramble or MAPT AON in presence of lipofectamine, immunostained for Tuj1 (grey) and cell nuclei were visualized by DAPI (blue). (**F-J**) Co-cultures transduced with EGFP-tagged $FTDtau^{1}$ or $FTDtau^{1+2}$ were treated at week 1 and 2 with 100nM MAPT or scramble AON in the presence of 0.5 µL lipofectamine per µg AON. Co-cultures were fixed at week 4, and tau levels and aggregation were assessed after 4 weeks. See *Materials and Methods* for more detail. (**F**) Schematic representation of the experiment. Representative confocal images of PFA-fixed (**G**) and MeOH-fixed (**I**) co-cultures treated with scramble of MAPT AON to visualize total levels and insoluble aggregates of EGFP-tagged $FTDtau^{1+2}$, respectively. EGFP direct fluorescence and immunostainings for MAP2 and a pathological conformation of tau (MC1) are shown in greyscale. Cell nuclei were visualized using DAPI (blue). Merged images include MAP2 (blue), $FTDtau^{1+2}$ (green) and MC1 (magenta). (**H** and **J**) Quantification of EGFP mean fluorescence intensity from **G** showing total tau levels and EGFP mean fluorescence standard deviation (SD) from **I** showing aggregated tau level, respectively, within a MAP2-positive mask in $FTDtau^{1}$- (grey) or $FTDtau^{1+2}$- (red) transduced co-cultures treated with scramble (-) and MAPT (+) AON. *N* = 3 independent experiment, *n* ≥ 16 wells. Bar graphs show the mean ± SEM, data points represent mean/well. Shapiro-Wilk normality testing followed by two-way ANOVA with Tukey's post-hoc analysis. **p<0.01, ****p<0.0001, *ns* not significant. Supplementary Table S1 lists the total number of cells analysed and exact p values.

Supplementary Figure S11

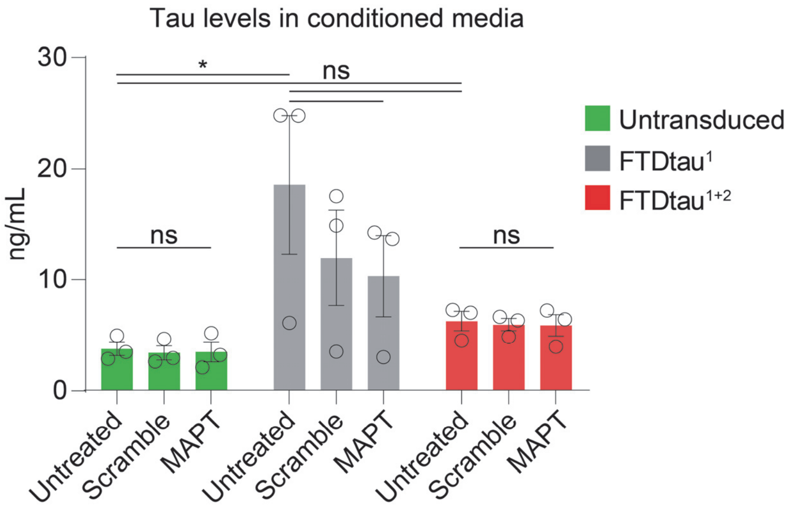

Extracellular tau levels are not different between FTDtau[1]- and FTDtau[1+2]-expressing co-cultures and are not affected by MAPT antisense treatment.

Total tau levels in conditioned media of untransduced co-cultures and co-cultures expressing EGFP- tagged FTDtau[1] or FTDtau[1+2], in the absence (untreated) or presence of scramble or MAPT AON treatment at week 4 and 6. After 8 weeks of co-culture, tau levels were measured using an MSD S-Plex Total Tau assay. Conditioned media from *N* = 3 independent experiments was pooled from *n* ≥ 3 wells per experiment. Bar graphs show the mean value per independent experiment ± SEM. Shapiro-Wilk normality testing followed by two-way ANOVA with Tukey's post-hoc analysis. *p<0.05, *ns* not significant. Supplementary Table S1 lists the exact p values.

Supplementary Table S1

Figure	Experiment	Experimental details	Indep. Exps.	Wells, outliers	# cells analysed		Statistical test	P value
					Neurons	Astrocytes		
Fig. 2C	Tau aggregation<br>week 4, 8	(1) FTDtau^{1}, week 4<br>(2) FTDtau^{1+2}, week 4<br>(3) FTDtau^{1}, week 8<br>(4) FTDtau^{1+2}, week 8	3<br>3<br>3<br>3	24<br>24<br>18<br>18	20,162<br>24,417<br>32,821<br>33,568		Two-way ANOVA<br>Tukey's Post Hoc	****p<0.0001: 1 versus 2, 2 versus 4, 3 versus 4<br>ns p=0.72: 1 versus 3
Fig. 2D	Dendrite length<br>week 4	FTDtau^{1}<br>FTDtau^{1+2}	17<br>17	64<br>64	173,639<br>195,233		Nested T test	ns p=0.22
Fig. 2E	Primary dendrites<br>week 4	FTDtau^{1}<br>FTDtau^{1+2}	17<br>17	64<br>64	173,639<br>195,233		Nested T test	ns p=0.18
Fig. 2F	Extremities<br>week 4	FTDtau^{1}<br>FTDtau^{1+2}	17<br>17	64<br>64	173,639<br>195,233		Nested T test	ns p=0.76
Fig. 2G	Segments<br>week 4	FTDtau^{1}<br>FTDtau^{1+2}	17<br>17	64<br>64	173,639<br>195,233		Nested T test	ns p=0.16
Fig. 2H	Branch points<br>week 4	FTDtau^{1}<br>FTDtau^{1+2}	17<br>17	64<br>64	173,639<br>195,233		Nested T test	*p=0.0275
Fig. 2I	Dendrite length<br>week 8	FTDtau^{1}<br>FTDtau^{1+2}	4<br>4	20<br>20	27,927<br>21,014		Nested T test	ns p=0.22
Fig. 2J	Primary dendrites<br>week 8	FTDtau^{1}<br>FTDtau^{1+2}	4<br>4	20<br>20	27,927<br>21,014		Nested T test	ns p=0.40
Fig. 2K	Extremities<br>week 8	FTDtau^{1}<br>FTDtau^{1+2}	4<br>4	20<br>20	27,927<br>21,014		Nested T test	ns p=0.63
Fig. 2L	Segments<br>week 8	FTDtau^{1}<br>FTDtau^{1+2}	4<br>4	20<br>20	27,927<br>21,014		Nested T test	ns p=0.75
Fig. 2M	Branch points<br>week 8	FTDtau^{1}<br>FTDtau^{1+2}	4<br>4	20<br>20	27,927<br>21,014		Nested T test	ns p=0.83
Fig. 2N	SYP1 density week 4	FTDtau^{1}<br>FTDtau^{1+2}	10<br>10	40<br>40	92,277<br>106,149		Nested T test	ns p=0.57
Fig. 2O	SYP1 intensity week 4	FTDtau^{1}<br>FTDtau^{1+2}	10<br>10	40<br>40	92,277<br>106,149		Nested T test	ns p=0.68
Fig. 2P	SYP1 density week 8	FTDtau^{1}<br>FTDtau^{1+2}	3<br>3	16<br>16	21,489<br>15,470		Nested T test	ns p=0.72
Fig. 2Q	SYP1 intensity week 8	FTDtau^{1}<br>FTDtau^{1+2}	3<br>3	16<br>16	21,489<br>15,470		Nested T test	ns p=0.67
Fig. 3D	Oxidative stress neurons<br>week 4	(1) FTDtau^{1} + Ctrl<br>(2) FTDtau^{1+2} + Ctrl<br>(3) FTDtau^{1} + Men<br>(4) FTDtau^{1+2} + Men	4<br>4<br>4<br>4	16<br>16<br>16<br>16	15,087<br>14,082<br>16,791<br>17,967		Two-way ANOVA<br>Tukey's Post Hoc	****p<0.0001: 1 versus 3, 2 versus 4<br>ns p=0.92: 1 versus 2, p=0.96: 3 versus 4
Fig. 3E	Oxidative stress neurons<br>week 8	(1) FTDtau^{1} + Ctrl<br>(2) FTDtau^{1+2} + Ctrl<br>(3) FTDtau^{1} + Men<br>(4) FTDtau^{1+2} + Men	4<br>4<br>4<br>4	14<br>14<br>14<br>14	14,582<br>18,985<br>19,145<br>24,921		Two-way ANOVA<br>Tukey's Post Hoc	****p<0.0001: 1 versus 3, 2 versus 4<br>ns p=0.83: 1 versus 2, p=0.75: 3 versus 4
Fig. 3F	Oxidative stress astrocytes<br>week 4	(1) FTDtau^{1} + Ctrl<br>(2) FTDtau^{1+2} + Ctrl<br>(3) FTDtau^{1} + Men<br>(4) FTDtau^{1+2} + Men	4<br>4<br>4<br>4	16<br>16<br>16<br>16		18,836<br>23,864<br>17,619<br>23,145	Two-way ANOVA<br>Tukey's Post Hoc	****p<0.0001: 1 versus 3, 2 versus 4<br>ns p=0.96: 1 versus 2, p=0.81: 3 versus 4
Fig. 3G	Oxidative stress astrocytes<br>week 8	(1) FTDtau^{1} + Ctrl<br>(2) FTDtau^{1+2} + Ctrl<br>(3) FTDtau^{1} + Men<br>(4) FTDtau^{1+2} + Men	4<br>4<br>4<br>4	14<br>14<br>14<br>14		14,500<br>14,805<br>8,409<br>8,651	Two-way ANOVA<br>Tukey's Post Hoc	****p<0.0001: 1 versus 3, 2 versus 4<br>***p=0.001: 1 versus 2<br>ns p=0.14: 3 versus 4
Fig. 4C	ISR activation<br>neurons week 4	(1) FTDtau^{1} + Ctrl<br>(2) FTDtau^{1+2} + Ctrl<br>(3) FTDtau^{1} + TM<br>(4) FTDtau^{1+2} + TM	3<br>3<br>3<br>3	12<br>12<br>12<br>12	44,499<br>36,818<br>41,515<br>33,751		Two-way ANOVA<br>Tukey's Post Hoc	****p<0.0001: 1 versus 3, 2 versus 4<br>ns p=0.98: 1 versus 2, p=0.74: 3 versus 4
Fig. 4D	ISR activation<br>neurons week 8	(1) FTDtau^{1} + Ctrl<br>(2) FTDtau^{1+2} + Ctrl<br>(3) FTDtau^{1} + TM<br>(4) FTDtau^{1+2} + TM	3<br>3<br>3<br>3	12<br>12<br>12<br>12	15,982<br>15,664<br>13,925<br>15,212		Two-way ANOVA<br>Tukey's Post Hoc	****p<0.0001: 1 versus 3, 2 versus 4<br>ns p=0.55: 1 versus 2, p=0.62: 3 versus 4
Fig. 4E	ISR activation<br>astrocytes week 4	(1) FTDtau^{1} + Ctrl<br>(2) FTDtau^{1+2} + Ctrl<br>(3) FTDtau^{1} + TM<br>(4) FTDtau^{1+2} + TM	3<br>3<br>3<br>3	12<br>12<br>12<br>12		28,040<br>24,357<br>29,648<br>24,050	Two-way ANOVA<br>Tukey's Post Hoc	****p<0.0001: 1 versus 3, 2 versus 4<br>ns p=0.96: 1 versus 2, p=0.98: 3 versus 4
Fig. 4F	ISR activation<br>astrocytes week 8	(1) FTDtau^{1} + Ctrl<br>(2) FTDtau^{1+2} + Ctrl<br>(3) FTDtau^{1} + TM<br>(4) FTDtau^{1+2} + TM	3<br>3<br>3<br>3	12<br>12<br>12<br>12		10,015<br>11,116<br>9,146<br>10,386	Two-way ANOVA<br>Tukey's Post Hoc	****p<0.0001: 1 versus 3, 2 versus 4.<br>***p=0.0002: 1 versus 2<br>***p=0.0004: 3 versus 4
Fig. 5C	AON therapy<br>Neuronal viability week 8	(1) Untransduced, scramble<br>(2) FTDtau^{1}, scramble<br>(3) FTDtau^{1+2}, scramble<br>(4) Untransduced, MAPT<br>(5) FTDtau^{1}, MAPT<br>(6) FTDtau^{1+2}, MAPT	4<br>7<br>7<br>4<br>7<br>7	16<br>40<br>40<br>16<br>40<br>40	14,994<br>30,355<br>31,312<br>16,908<br>24,327<br>32,335	6,396<br>13,872<br>15,977<br>7,227<br>10,779<br>14,976	Two-way ANOVA<br>Tukey's Post Hoc	ns p>0.99: 1 versus 4, 5 versus 6, p=0.56: 1 versus 2, p=0.90: 2 versus 3, p=0.91: 4 versus 5, 4 versus 6
Fig. 5E	AON therapy<br>Tau levels week 8	(1) FTDtau^{1} + Scramble<br>(2) FTDtau^{1} + MAPT<br>(3) FTDtau^{1+2} + Scramble<br>(4) FTDtau^{1+2} + MAPT	2<br>2<br>2<br>2	11, 1<br>12<br>12<br>12	4,639<br>4,525<br>5,578<br>5,254		Two-way ANOVA<br>Tukey's Post Hoc	****p<0.0001: 1 versus 2, 3 versus 4<br>ns p=0.54: 1 versus 3, p=0.89: 2 versus 4
Fig. 5G	AON therapy<br>Tau aggregation week 8	(1) FTDtau^{1} + Scramble<br>(2) FTDtau^{1} + MAPT<br>(3) FTDtau^{1+2} + Scramble<br>(4) FTDtau^{1+2} + MAPT	4<br>4<br>4<br>4	26<br>26<br>26<br>26	20,624<br>23,488<br>17,498<br>20,053		Two-way ANOVA<br>Tukey's Post Hoc	****p<0.0001: 1 versus 3, 2 versus 4<br>**p=0.002: 3 versus 4<br>ns p=0.97: 1 versus 2
Fig. 6B	AON therapy<br>Oxidative stress<br>neurons week 8	(1) FTDtau^{1} + Scramble<br>(2) FTDtau^{1} + MAPT<br>(3) FTDtau^{1+2} + Scramble<br>(4) FTDtau^{1+2} + MAPT	4<br>4<br>4<br>4	16<br>15, 1<br>16<br>16	8,604<br>10,616<br>12,934<br>14,001		Two-way ANOVA<br>Tukey's Post Hoc	ns p=0.86: 1 versus 2, p=0.88: 1 versus 3<br>p=0.62: 2 versus 4, p=0.57: 3 versus 4

Figure	Experiment	Experimental details	Indep. Exps.	Wells, outliers	# cells analysed		Statistical test	P value
					Neurons	Astrocytes		
Fig. 6C	AON therapy<br>Oxidative stress astrocytes<br>week 8	(1) FTDtau^{1} + Scramble<br>(2) FTDtau^{1} + MAPT<br>(3) FTDtau^{1+2} + Scramble<br>(4) FTDtau^{1+2} + MAPT	4<br>4<br>4<br>4	16<br>16<br>16<br>15, 1		4,907<br>5,320<br>7,257<br>6,847	Two-way ANOVA<br>Tukey's Post Hoc	****p<0.0001: 1 versus 3<br>*p=0.024: 3 versus 4<br>ns p=0.97: 1 versus 2, p=0.34: 2 versus 4
Fig. 6D	AON therapy<br>ISR activation<br>neurons week 8	(1) FTDtau^{1} + Scramble<br>(2) FTDtau^{1} + MAPT<br>(3) FTDtau^{1+2} + Scramble<br>(4) FTDtau^{1+2} + MAPT	4<br>4<br>4<br>4	15<br>15<br>14<br>14	7,404<br>9,103<br>6,852<br>9,431		Two-way ANOVA<br>Tukey's Post Hoc	ns p=0.76: 1 versus 2, p=0.94: 1 versus 3<br>p=0.65: 2 versus 4, p=0.051: 3 versus 4
Fig. 6E	AON therapy<br>ISR activation<br>astrocytes week 8	(1) FTDtau^{1} + Scramble<br>(2) FTDtau^{1} + MAPT<br>(3) FTDtau^{1+2} + Scramble<br>(4) FTDtau^{1+2} + MAPT	4<br>4<br>4<br>4	15<br>15<br>14<br>14		3,905<br>4,820<br>3,845<br>5,366	Two-way ANOVA<br>Tukey's Post Hoc	****p<0.0001: 1 versus 3<br>**p=0.001: 3 versus 4<br>ns p=0.78: 1 versus 2, p=0.0.071: 2 versus 4
Sup. Fig. S2B	Neuronal viability week 4	Untransduced<br>FTDtau^{1}	4<br>4	16<br>16	43,030<br>41,464	15,507<br>15,933	Nested T test	ns p=0.98
Sup. Fig. S2C	Dendrite length week 4	Untransduced<br>FTDtau^{1}	4<br>4	16<br>16	43,030<br>41,464		Nested T test	ns p=0.99
Sup. Fig. S2D	Primary dendrites week 4	Untransduced<br>FTDtau^{1}	4<br>4	16<br>16	43,030<br>41,464		Nested T test	ns p=0.59
Sup. Fig. S2E	Extremities week 4	Untransduced<br>FTDtau^{1}	4<br>4	16<br>16	43,030<br>41,464		Nested T test	ns p=0.83
Sup. Fig. S2F	Segments week 4	Untransduced<br>FTDtau^{1}	4<br>4	16<br>16	43,030<br>41,464		Nested T test	ns p=0.84
Sup. Fig. S2G	Branch points week 4	Untransduced<br>FTDtau^{1}	4<br>4	16<br>16	43,030<br>41,464		Nested T test	ns p=0.90
Sup. Fig. S2H	SYP1 density week 4	Untransduced<br>FTDtau^{1}	4<br>4	16<br>16	43,030<br>41,464		Nested T test	ns p=0.84
Sup. Fig. S2I	SYP1 intensity week 4	Untransduced<br>FTDtau^{1}	4<br>4	16<br>16	43,030<br>41,464		Nested T test	ns p=0.87
Sup. Fig. S4D	Fluorescence SD : MC1	FTDtau^{1+2}	4	34	45,219		Pearson correlation	****p<0.0001, r = 0.95, R^2 = 0.90
Sup. Fig. S5C	Tau levels<br>week 1, 2½, 4	(1) Untransduced, week 1<br>(2) FTDtau^{1}, week 1<br>(3) FTDtau^{1+2}, week 1<br>(4) Untransduced, week 2½<br>(5) FTDtau^{1}, week 2½<br>(6) FTDtau^{1+2}, week 2½<br>(7) Untransduced, week 4<br>(8) FTDtau^{1}, week 4<br>(9) FTDtau^{1+2}, week 4	3<br>3<br>3<br>3<br>3<br>3<br>3<br>3<br>3	12<br>12<br>12<br>12<br>12<br>12<br>12<br>12<br>12	5,255<br>5,392<br>5,355<br>17,781<br>15,498<br>12,443<br>19,413<br>16,041<br>13,778		Two-way ANOVA<br>Tukey's Post Hoc	****p<0.0001: 5 versus 6, 8 versus 9, 3 versus 6, 6 versus 9<br>***p=0.0002: 6 versus 9<br>*p=0.0152: 5 versus 8<br>ns p=0.56: 8 versus 9, p>0.99: 1 versus 2, 2 versus 3, 1 versus 3, 5 versus 6
Sup. Fig. S5E	Tau aggregation<br>week 1, 2½, 4	(1) Untransduced, week 1<br>(2) FTDtau^{1}, week 1<br>(3) FTDtau^{1+2}, week 1<br>(4) Untransduced, week 2½<br>(5) FTDtau^{1}, week 2½<br>(6) FTDtau^{1+2}, week 2½<br>(7) Untransduced, week 4<br>(8) FTDtau^{1}, week 4<br>(9) FTDtau^{1+2}, week 4	3<br>3<br>3<br>3<br>3<br>3<br>1<br>2<br>2	12<br>12<br>11, 1<br>12<br>12<br>12<br>4<br>8<br>8	6,529<br>7,148<br>6,787<br>14,285<br>9,501<br>9,149<br>10,203<br>10,606<br>8,196		Two-way ANOVA<br>Tukey's Post Hoc	****p<0.0001: 5 versus 6, 8 versus 9, 3 versus 6, 6 versus 9<br>ns p>0.99: 1 versus 2, 2 versus 3, 1 versus 3, 2 versus 5, 4 versus 5, 5 versus 8, 7 versus 8.
Sup. Fig. S6B	Dendrite length<br>week 1, 2, 3, 4	(1) FTDtau^{1}, week 1<br>(2) FTDtau^{1+2}, week 1<br>(3) FTDtau^{1}, week 2<br>(4) FTDtau^{1+2}, week 2<br>(5) FTDtau^{1}, week 3<br>(6) FTDtau^{1+2}, week 3<br>(7) FTDtau^{1}, week 4<br>(8) FTDtau^{1+2}, week 4	5<br>5<br>4<br>4<br>4<br>4<br>5<br>5	20<br>20<br>16<br>16<br>16<br>16<br>20<br>20	18,782<br>22,427<br>23,811<br>28,623<br>31,637<br>37,376<br>37,700<br>44,097		Regression<br>&<br>Two-way ANOVA<br>Tukey's Post Hoc	Regression values in Sup. Fig. S 6G.<br>*p=0.043: 2 versus 8<br>*p=0.012: 3 versus 7<br>ns p=0.25: 1 versus 7, p=0.66: 1 versus 2, p>0.99: 3 versus 4, 5 versus 6, 7 versus 8
Sup. Fig. S6C	Primary dendrites<br>week 1, 2, 3, 4	(1) FTDtau^{1}, week 1<br>(2) FTDtau^{1+2}, week 1<br>(3) FTDtau^{1}, week 2<br>(4) FTDtau^{1+2}, week 2<br>(5) FTDtau^{1}, week 3<br>(6) FTDtau^{1+2}, week 3<br>(7) FTDtau^{1}, week 4<br>(8) FTDtau^{1+2}, week 4	5<br>5<br>4<br>4<br>4<br>4<br>5<br>5	20<br>20<br>16<br>16<br>16<br>16<br>20<br>20	18,782<br>22,427<br>23,811<br>28,623<br>31,637<br>37,376<br>37,700<br>44,097		Regression<br>&<br>Two-way ANOVA<br>Tukey's Post Hoc	Regression values in Sup. Fig. S 6G.<br>****p<0.0001: 1 versus 3<br>***p=0.0004: 2 versus 4<br>**p=0.009: 3 versus 7<br>ns p=0.49: 3 versus 4, p=0.50: 2 versus 8, p=0.88: 1 versus 2, p=0.96: 1 versus 7, p>0.99: 5 versus 6, 7 versus 8
Sup. Fig. S6D	Extremities<br>week 1, 2, 3, 4	(1) FTDtau^{1}, week 1<br>(2) FTDtau^{1+2}, week 1<br>(3) FTDtau^{1}, week 2<br>(4) FTDtau^{1+2}, week 2<br>(5) FTDtau^{1}, week 3<br>(6) FTDtau^{1+2}, week 3<br>(7) FTDtau^{1}, week 4<br>(8) FTDtau^{1+2}, week 4	5<br>5<br>4<br>4<br>4<br>4<br>5<br>5	20<br>20<br>16<br>16<br>16<br>16<br>20<br>20	18,782<br>22,427<br>23,811<br>28,623<br>31,637<br>37,376<br>37,700<br>44,097		Regression<br>&<br>Two-way ANOVA<br>Tukey's Post Hoc	Regression values in Sup. Fig. S 6G.<br>****p<0.0001: 1 versus 5, 1 versus 7, 2 versus 6, 2 versus 8<br>***p=0.0008: 2 versus 4<br>*p=0.015: 1 versus 3<br>ns p>0.99: 1 versus 2, 3 versus 4, 5 versus 6, 7 versus 8.
Sup. Fig. S6E	Segments<br>week 1, 2, 3, 4	(1) FTDtau^{1}, week 1<br>(2) FTDtau^{1+2}, week 1<br>(3) FTDtau^{1}, week 2<br>(4) FTDtau^{1+2}, week 2<br>(5) FTDtau^{1}, week 3<br>(6) FTDtau^{1+2}, week 3<br>(7) FTDtau^{1}, week 4<br>(8) FTDtau^{1+2}, week 4	5<br>5<br>4<br>4<br>4<br>4<br>5<br>5	20<br>20<br>16<br>16<br>16<br>16<br>20<br>20	18,782<br>22,427<br>23,811<br>28,623<br>31,637<br>37,376<br>37,700<br>44,097		Regression<br>&<br>Two-way ANOVA<br>Tukey's Post Hoc	Regression values in Sup. Fig. S 6G.<br>****p<0.0001: 1 versus 5, 1 versus 7, 2 versus 6, 2 versus 8.<br>*p=0.035: 1 versus 3.<br>*p=0.015: 2 versus 4<br>ns p>0.99: 1 versus 2, 3 versus 4, 5 versus 6, 7 versus 8.

Figure	Experiment	Experimental details	Indep. Exps.	Wells, outliers	# cells analysed		Statistical test	P value
					Neurons	Astrocytes		
Sup. Fig. S6F	Branch points<br>week 1, 2, 3, 4	(1) FTDtau^{1}, week 1<br>(2) FTDtau^{1+2}, week 1<br>(3) FTDtau^{1}, week 2<br>(4) FTDtau^{1+2}, week 2<br>(5) FTDtau^{1}, week 3<br>(6) FTDtau^{1+2}, week 3<br>(7) FTDtau^{1}, week 4<br>(8) FTDtau^{1+2}, week 4	5<br>5<br>4<br>4<br>4<br>4<br>5<br>5	20<br>20<br>16<br>16<br>16<br>16<br>20<br>20	18,782<br>22,427<br>23,811<br>28,623<br>31,637<br>37,376<br>37,700<br>44,097		Regression<br>&<br>Two-way ANOVA<br>Tukey's Post Hoc	Regression values in Sup. Fig. S 6G.<br>****p<0.0001: 1 versus 5, 1 versus 7, 2 versus 6, 2 versus 8<br>*p=0.011: 2 versus 4<br>*p=0.040: 1 versus 3<br>ns p>0.99: 1 versus 2, 3 versus 4, 5 versus 6, 7 versus 8.
Sup. Fig. S8A	Oxidative stress neurons<br>week 8	(1) Untransduced + Ctrl<br>(2) FTDtau^{1} + Ctrl<br>(3) Untransduced + Men<br>(4) FTDtau^{1} + Men	3<br>3<br>3<br>3	12<br>12<br>12<br>12	18,239<br>12,774<br>24,536<br>17,346		Two-way ANOVA<br>Tukey's Post Hoc	****p<0.0001: 1 versus 3, 2 versus 4<br>ns p=0.89: 1 versus 2, p=0.52: 3 versus 4
Sup. Fig. S8B	Oxidative stress astrocytes<br>week 8	(1) Untransduced + Ctrl<br>(2) FTDtau^{1} + Ctrl<br>(3) Untransduced + Men<br>(4) FTDtau^{1} + Men	3<br>3<br>3<br>3	12<br>12<br>12<br>12		14,172<br>13,228<br>8,722<br>7,586	Two-way ANOVA<br>Tukey's Post Hoc	****p<0.0001: 1 versus 3, 2 versus 4<br>ns p=0.47: 1 versus 2, p=0.39: 3 versus 4
Sup. Fig. S8C	ISR activation<br>neurons week 8	(1) Untransduced + Ctrl<br>(2) FTDtau^{1} + Ctrl<br>(3) Untransduced + TM<br>(4) FTDtau^{1} + TM	1<br>1<br>1<br>1	4<br>4<br>4<br>4	8,454<br>6,956<br>8,682<br>6,171		Two-way ANOVA<br>Tukey's Post Hoc	****p<0.0001: 1 versus 3, 2 versus 4<br>ns p=0.57: 1 versus 2, p=0.09: 3 versus 4
Sup. Fig. S8D	ISR activation<br>astrocytes week 8	(1) Untransduced + Ctrl<br>(2) FTDtau^{1} + Ctrl<br>(3) Untransduced + TM<br>(4) FTDtau^{1} + TM	1<br>1<br>1<br>1	4<br>4<br>4<br>4		5,118<br>3,835<br>4,679<br>3,325	Two-way ANOVA<br>Tukey's Post Hoc	****p<0.0001: 1 versus 3, 2 versus 4<br>ns p=0.97: 1 versus 2, p=0.13: 3 versus 4
Sup. Fig. S9C	Oxidative stress<br>monocultured astrocytes	Untransduced + Ctrl<br>Untransduced + Men	1<br>1	4<br>4		907<br>809	Student's T test	****p<0.0001
Sup. Fig. S9E	ISR activation<br>monocultured astrocytes	Untransduced + Ctrl<br>Untransduced + TM	1<br>1	4<br>4		907<br>500	Student's T test	***p=0.0001
Sup. Fig. S9G	Oxidative stress<br>conditioned media	(1) Untransduced<br>(2) FTDtau^{1}<br>(3) FTDtau^{1+2}	3<br>3<br>3	6<br>6<br>6		1,638<br>1,500<br>1,422	Nested One-Way<br>ANOVA	ns p=0.21: 2 versus 3, p=0.38: 1 versus 2, p=0.88: 1 versus 3
Sup. Fig. S9I	ISR activation<br>conditioned media	(1) Untransduced<br>(2) FTDtau^{1}<br>(3) FTDtau^{1+2}	3<br>3<br>3	6<br>6<br>6		1,638<br>1,500<br>1,422	Nested One-Way<br>ANOVA	ns p=0.25: 1 versus 2, p=0.62: 1 versus 3, p=0.76: 2 versus 3
Sup. Fig. S10C	AON delivery	(1) Untreated<br>(2) Lipo<br>(3) AON<br>(4) Lipo + AON	1<br>1<br>1<br>1	3<br>3<br>3<br>3	1,708<br>1,778<br>1,977<br>1,522		Two-way ANOVA<br>Tukey's Post Hoc	****p<0.0001: 2 versus 4, 3 versus 4<br>***p=0.0001: 1 versus 3<br>ns p=0.98: 1 versus 2
Sup. Fig. S10H	AON therapy<br>Tau levels week 4	(1) FTDtau^{1} + Scramble<br>(2) FTDtau^{1} + MAPT<br>(3) FTDtau^{1+2} + Scramble<br>(4) FTDtau^{1+2} + MAPT	3<br>3<br>3<br>3	16<br>16<br>16<br>16	8,269<br>5,836<br>7,709<br>6,321		Two-way ANOVA<br>Tukey's Post Hoc	****p<0.0001: 1 versus 2<br>**p=0.0081: 3 versus 4<br>ns p=0.08: 1 versus 3, p=0.84: 2 versus 4
Sup. Fig. S10J	AON therapy<br>Tau aggregation week 4	(1) FTDtau^{1} + Scramble<br>(2) FTDtau^{1} + MAPT<br>(3) FTDtau^{1+2} + Scramble<br>(4) FTDtau^{1+2} + MAPT	3<br>3<br>3<br>3	18<br>18<br>18<br>18	14,561<br>11,327<br>16,855<br>14,383		Two-way ANOVA<br>Tukey's Post Hoc	****p<0.0001: 1 versus 3, 3 versus 4<br>ns p=0.11: 2 versus 4
Sup. Fig. S11	Tau levels<br>conditioned media	(1) Untransduced, untreated<br>(2) Untransduced + Scramble<br>(3) Untransduced + MAPT<br>(4) FTDtau^{1}, untreated<br>(5) FTDtau^{1} + Scramble<br>(6) FTDtau^{1} + MAPT<br>(7) FTDtau^{1+2}, untreated<br>(8) FTDtau^{1+2} + Scramble<br>(9) FTDtau^{1+2} + MAPT	3<br>3<br>3<br>3<br>3<br>3<br>3<br>3<br>3				Two-way ANOVA<br>Tukey's Post Hoc	*p<0.0001: 1 versus 4<br>ns p=0.13: 4 versus 7, ns p=0.78: 4 versus 5,<br>ns p>0.99: 1 versus 2, 1 versus 3, 5 versus 6, 7 versus 8, 8 versus 9

Supplementary Table 1

Overview of the number of independent experiments/wells/cells, statistical tests and exact p values.

Table showing experimental and statistical details for each dataset in this study.
